# Genomic epidemiology reveals multidrug resistant plasmid spread between *Vibrio cholerae* lineages in Yemen

**DOI:** 10.1038/s41564-023-01472-1

**Published:** 2023-09-28

**Authors:** Florent Lassalle, Salah Al-Shalali, Mukhtar Al-Hakimi, Elisabeth Njamkepo, Ismail Mahat Bashir, Matthew J. Dorman, Jean Rauzier, Grace A. Blackwell, Alyce Taylor-Brown, Mathew A. Beale, Adrián Cazares, Ali Abdullah Al-Somainy, Anas Al-Mahbashi, Khaled Almoayed, Mohammed Aldawla, Abdulelah Al-Harazi, Marie-Laure Quilici, François-Xavier Weill, Ghulam Dhabaan, Nicholas R. Thomson

**Affiliations:** 1 Parasites and Microbes Programme, Wellcome Sanger Institute, Hinxton, UK; 2 Faculty of Science, Sana’a University, Sana’a, Yemen; 3 Institut Pasteur, Université Paris Cité, Unité des Bactéries pathogènes entériques, Paris, France; 4 WHO Yemen country office, Sana’a, Yemen; 5 Churchill College, Cambridge, UK; 6 EMBL-EBI, Hinxton, UK; 7 National Centre of Public Health Laboratories, Sana’a, Yemen; 8 Ministry of Public Health, Infection Control Unit, Sana’a, Yemen; 9 Department of Laboratory Medicine and Pathobiology, University of Toronto, Toronto, Ontario Canada; 10 London School of Hygiene and Tropical Medicine, London, UK

**Keywords:** Pathogens, Genome evolution, Molecular evolution

## Abstract

Since 2016, Yemen has been experiencing the largest cholera outbreak in modern history. Multidrug resistance (MDR) emerged among *Vibrio cholerae* isolates from cholera patients in 2018. Here, to characterize circulating genotypes, we analysed 260 isolates sampled in Yemen between 2018 and 2019. Eighty-four percent of *V. cholerae* isolates were serogroup O1 belonging to the seventh pandemic El Tor (7PET) lineage, sub-lineage T13, whereas 16% were non-toxigenic, from divergent non-7PET lineages. Treatment of severe cholera with macrolides between 2016 and 2019 coincided with the emergence and dominance of T13 subclones carrying an incompatibility type C (IncC) plasmid harbouring an MDR pseudo-compound transposon. MDR plasmid detection also in endemic non-7PET *V. cholerae* lineages suggested genetic exchange with 7PET epidemic strains. Stable co-occurrence of the IncC plasmid with the SXT family of integrative and conjugative element in the 7PET background has major implications for cholera control, highlighting the importance of genomic epidemiological surveillance to limit MDR spread.

## Abstract (web summary)

Genomic epidemiology of *Vibrio cholerae* isolates recovered between 2016 and 2019 during the Yemen cholera outbreak reveals acquisition of multidrug resistance and patterns of plasmid transmission between endemic and epidemic lineages.

## Main

Since 2016, Yemen has seen the largest epidemic of cholera ever recorded. This occurred against the backdrop of a civil war turned international conflict and famine, which together fuelled extensive population movement, with more than four million people internally displaced by the end of 2020^1^. The Electronic Disease Early Warning System, a surveillance programme coordinated by the Ministry of Public Health and Population of Yemen (MPHP) in Sana’a tasked with monitoring the epidemic^2^, recorded a total of almost 2.4 million suspected cholera cases up until August 2019^3^. These cases exhibited a seasonal profile, with peaks in July 2017 and September 2018 (16,000 and 50,000 cases per week, respectively)^3^. The lower reported case incidence in 2018 was ascribed to the mass vaccination campaign led by the World Health Organization and United Nation Children’s Fund, who delivered the oral cholera vaccine to 540,000 people in August 2018 (and 387,000 at follow-up in September) in targeted districts in Aden, Hudaydah and Ibb governorates^4,5^. Despite this mass vaccination campaign, cholera cases were recorded nationwide in 2019, peaking at over 30,000 cases per week and case numbers declined at a slower rate than in previous years^3^.

Pandemic cholera is caused by specific phylogenetic lineages of the bacterium *Vibrio cholerae* that are associated with epidemic spread, and which carry lipopolysaccharide O-antigens of serogroups O1 or O139. The large majority of epidemic strains associated with cholera outbreaks from the past 60 years belong to the seventh pandemic El Tor (7PET) lineage of *V. cholerae* O1, which has spread globally in three pandemic waves^6^. We previously used genomic epidemiology to show that the first two waves of the cholera outbreak in Yemen (2016 and 2017) were driven by a single clonal expansion^7^ belonging to Wave 3 of the global 7PET lineage and had an Ogawa serotype. This indicated the Yemen outbreak was seeded by a single international transmission event linked to the 7PET sub-lineage denoted T13 (ref. ^7^).

Ongoing surveillance activities in Yemen found that the fluctuating peaks in incidence were accompanied by a sudden change in the antibiotic susceptibility profile reported by the reference laboratory at the MPHP in Sana’a. Although strains isolated in 2016–2018 were sensitive to most of the antibiotics usually used for the treatment of cholera (except quinolones, where reduced susceptibility to ciprofloxacin prevented the use of this antibiotic as a single-dose treatment), by 2019, resistance was observed for multiple drugs including third-generation cephalosporins, macrolides (including azithromycin) and co-trimoxazole. Although the main treatment for cholera is rehydration therapy, antibiotics can be used to limit the volume and duration of the acute watery diarrhoea, and reduce the risk of transmission^8–10^. In Yemen, macrolides were used extensively up to early 2019 to treat moderate to severe cases of cholera in pregnant women and children, the latter forming the large majority of cases^11^. Multiple drug resistance (MDR) in *V. cholerae* is strongly associated with the acquisition of mobile genetic elements (MGEs) such as SXT family integrative and conjugative elements (SXT ICE) or plasmids of the incompatibility type C (IncC; formerly known as IncA/C_2_)^12^, which often carry and disseminate antimicrobial resistance (AMR) gene cargo^13^.

We hypothesized that the MDR phenotype seen in the Yemen *V. cholerae* isolates from 2019 could be explained either by gain of resistance (through de novo mutations or acquisition of a resistance-conferring MGE) in the previously susceptible 7PET-T13 *V. cholerae* strain already circulating in Yemen, or through the replacement of that strain with locally or globally derived MDR strain(s). Distinguishing between these hypotheses is important for understanding the ongoing dynamics of cholera in Yemen and will be important for cholera control strategies. We therefore applied genomic epidemiology approaches to determine the molecular basis for the observed switch to the MDR phenotype and its link to evolutionary dynamics of pandemic cholera. In doing so, we highlight the role of globally circulating MGEs in making an epidemic pathogen resistant to multiple drugs and subsequently reducing treatment options. We also show that these MGEs and their cargo AMR genes were repeatedly exchanged among diverse *V. cholerae* lineages found in Yemen.

## Results

### *V. cholerae* in Yemen in 2018 and 2019

**Extended Data Fig. 1:**
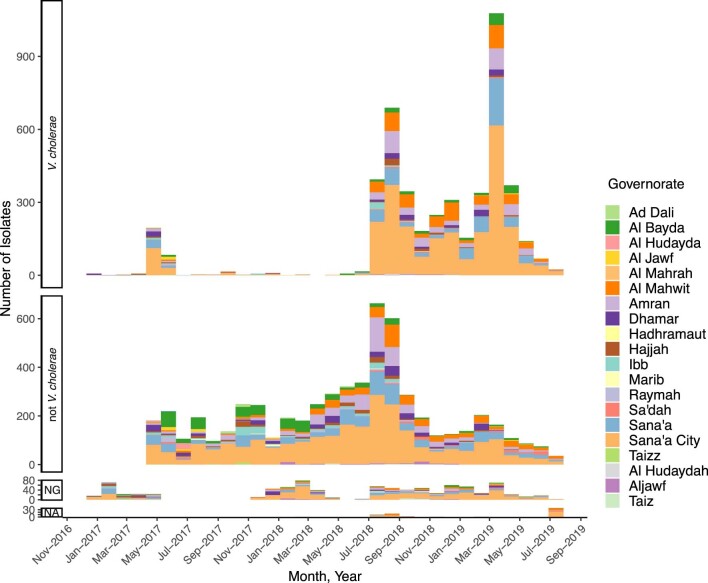
Culture confirmation of samples derived from suspected cholera cases in Yemen, 2017–2019. Distribution over time of *V. cholerae* culture result samples received at the NCPHL, broken down by governorate. Data are derived from Electronic Disease Early Warning System (eDEWS) sample lists (Supplementary Table 1). NG, no growth; NA, not available.

**Extended Data Fig. 2:**
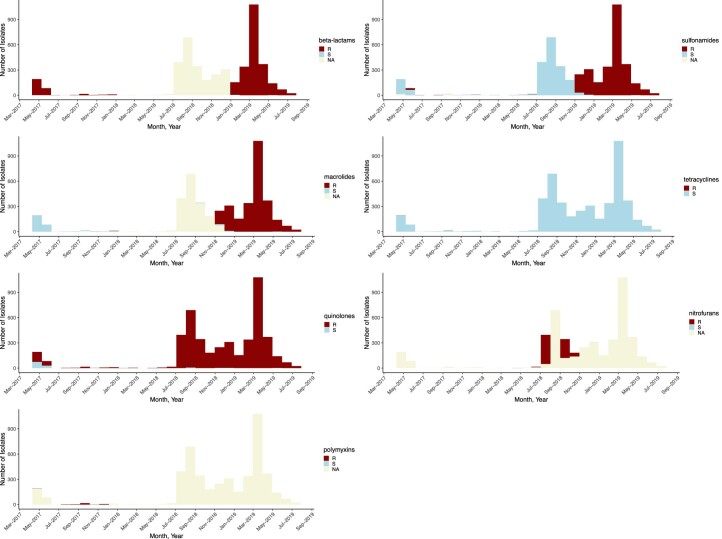
Antibiotic susceptibility phenotypes of all *V. cholerae* isolates collected in Yemen, 2017 and 2019. Distribution over time of resistance and sensitivity to broad antibiotic classes among culture-confirmed *V. cholerae* isolates received at the NCPHL. Data are derived from eDEWS sample lists (Supplementary Table 1).

**Extended Data Fig. 3:**
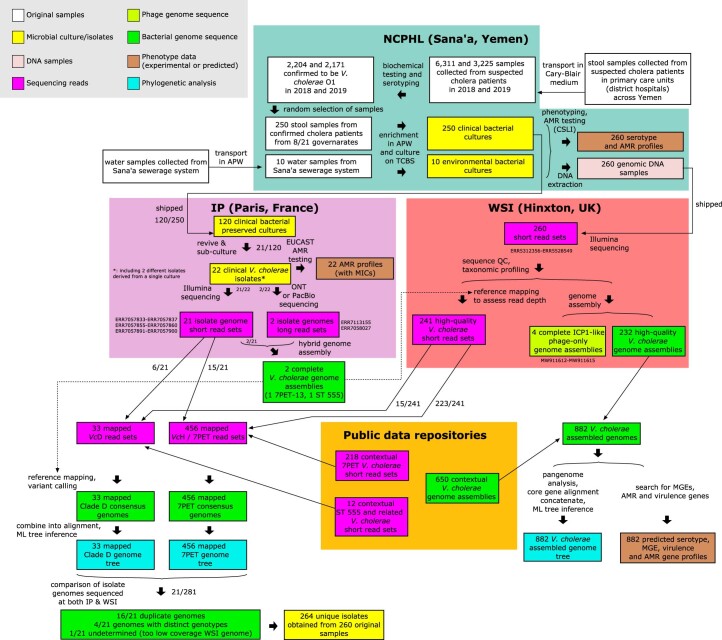
Flowchart of sample collection, management and use in protocols and analyses. Experiments and analyses are itemized and grouped according to the different locations of the collaborative consortium where they were undertaken: NCPHL, The National Centre of Public Health Laboratories; IP, Institut Pasteur; WSI, Wellcome Sanger Institute.

The National Centre of Public Health Laboratories (NCPHL) in Sana’a, the capital city of Yemen, received 6,311 and 3,225 clinical samples from suspected cholera patients in 2018 and 2019, respectively (Supplementary Table 1). Of these, 2,204 (35%) and 2,171 (67%) were confirmed to be positive for *V. cholerae* O1 by culture (identification based on biochemical tests and detection of Ogawa and Inaba serotypes) (Supplementary Table 1 and Extended Data Fig. 1). Among the 1,642 *V. cholerae* isolated at the NCPHL from January to October 2018, 623 were tested for susceptibility to a range of antibiotics using the disk diffusion method, of these 620 (99.6%) were phenotypically resistant to nalidixic acid and nitrofurantoin, but otherwise sensitive to all other antimicrobials tested (Extended Data Fig. 2 and Supplementary Table 1). By contrast, all tested *V. cholerae* isolates (*n* = 2,172) from January 2019 onwards were resistant to nalidixic acid, azithromycin, co-trimoxazole and cefotaxime (Supplementary Fig. 2 and Supplementary Table 1), a pattern maintained up to late 2021 (see ref. ^14^ for 2020–2021 cholera surveillance data). The transition in phenotype occurred during November 2018, when 159/175 (90.8%) tested isolates already showed the MDR profile. Of the 2018–2019 clinical *V. cholerae* isolates, 250 were randomly chosen for further characterization (Supplementary Table 2). These samples originated from 8 of the 21 Yemen governorates, comprising 71 of 333 districts, with 101 samples collected in 2018 (from mid-July to late October) and 149 collected in 2019 (from late February to late April and from early August to mid-October). In addition, ten environmentally derived strains were isolated from sewage in Sana’a in October 2019 (Supplementary Table 2). Extended antibiotic sensitivity testing of these 260 isolates at NCPHL and of a subset (*n* = 22) at Institut Pasteur (IP) (Extended Data Fig. 3) confirmed the phenotypic switch to MDR observed in the wider sample set, further showing that all tested 2019 strains were resistant to ampicillin, cefotaxime, nalidixic acid, azithromycin, erythromycin and co-trimoxazole (Supplementary Text).

### Phylogenetic diversity of *V. cholerae* in Yemen (2018, 2019)

**Fig. 1:**
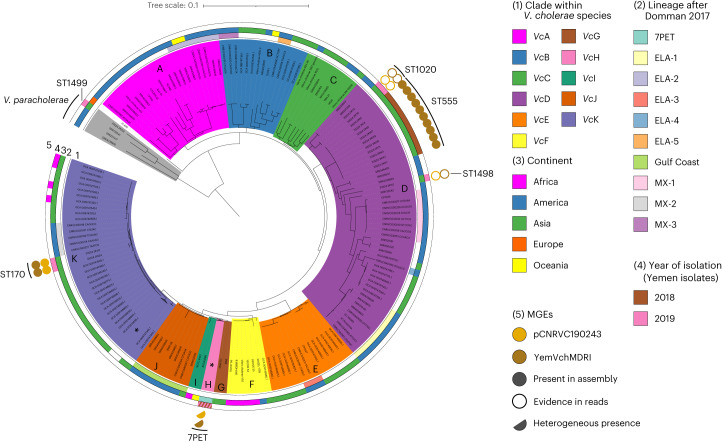
Phylogenetic diversity of *V. cholerae* isolates from Yemen. ML phylogeny of 882 assembled *V. cholerae* genomes based on the 37,170 SNP sites from the concatenated alignments of 291 core genes. Low-diversity clades (*Vc*H and part of *Vc*K) are collapsed and marked by black stars. Clades are highlighted with background colours (legend key 1). Coloured rings outside the tree depict the match with previously described lineages (ring 2), the geographical origin of isolates at the level of continents (ring 3) and their year of isolation when from Yemen (ring 4). The presence of parts of the plasmid pCNRVC190243 are indicated by coloured circles (ring 5 in A): IncC plasmid backbone (light brown) and the MDR PCT Yem*Vch*MDRI (dark brown); full circles indicate more than 70% coverage in assemblies of the reference length, hollow circles indicate 30%–70% coverage in assemblies and confirmed presence based on mapped reads, with even coverage over the MGE reference sequence, whereas half-circles represent heterogeneous presence in a collapsed clade. The scale bar represents the number of nucleotide substitutions per site.

**Extended Data Fig. 4:**
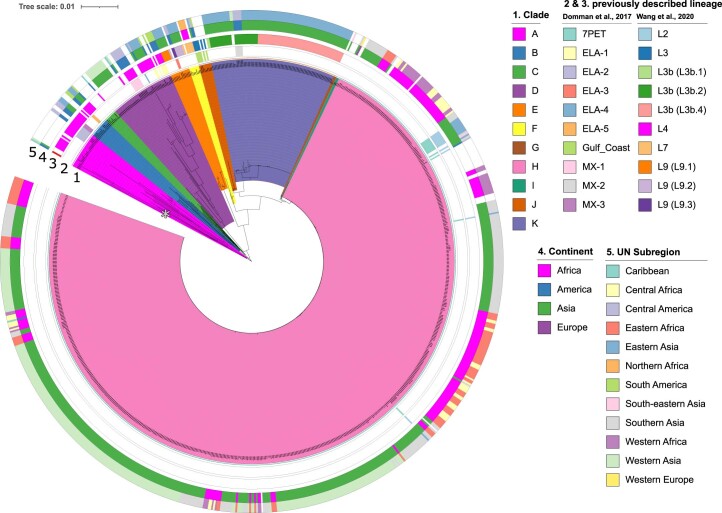
Phylogenetic diversity of *Vibrio cholerae* isolates from Yemen and contextual samples. Expanded version of phylogentic tree shown in Fig. 1 (no clades are collapsed).

We isolated a single colony for 240 of the 260 *V. cholerae* isolates indicated above, and multiple independent colony picks for the remaining 20, for a total of 281 isolates on which we performed whole-genome sequencing (Extended Data Fig. 3 and Supplementary Tables 2 and 3). After quality filtering, 232 high-quality isolate genomes were assembled (selecting a single isolate from each initial sample) (Supplementary Table 4), which we combined with 650 previously published *V. cholerae* O1 and non-O1 genomes for context (Supplementary Table 5 and Extended Data Fig. 3). We inferred a core-genome phylogeny for this genome set (*n* = 882), which described the sequenced diversity of the *V. cholerae* species, rooted by the genomes that belong to its newly described sister species *V. paracholerae*^15^. We subdivided *V. cholerae* genomes into 11 clades, referred to henceforth as *Vc*A to *Vc*K (Fig. 1 and Supplementary Table 5). *Vc*H contained all 7PET epidemic lineage genomes utilized in this dataset, including the majority (216/232) of the Yemen 2018–2019 genomes and all 42 previously reported 2016–2017 Yemeni genomes^7^ (Extended Data Fig. 4).

Although Yemeni *Vc*H isolates show limited genomic diversity (99.98%–100.00% average nucleotide identity (ANI) similarity; 0 to 97 single nucleotide polymorphisms (SNPs)), the remaining 16 Yemeni genomes belonged to clades *V. paracholerae* (*Vpc*), *Vc*D and *Vc*K, and were overall more diverse than *Vc*H isolate genomes (96.24%–99.99% ANI similarity) (Fig. 1 and Table 1); these represent ‘non-7PET’ lineages. Among these, we found five distinct sequence types in three lineages: *Vpc* (*n* = 1; ST1499), *Vc*D (*n* = 21; ST555, ST1020 and ST1498) (Supplementary Table 5) and *Vc*K (*n* = 2; ST170) (Fig. 1). Collectively, these non-7PET isolates comprised 8% of the clinical isolates (21/254) and 30% of environmentally derived isolates (3/10).

**Table 1:** Number of *V. cholerae* isolate genomes from Yemen by year and phylogenetic lineage

Year	Total	Clades				Clusters				Not determined^d^
		Non-7PET			7PET	H.9.e	H.9.f	H.9.g	H.9.h	
		*Vpc*^a^	*Vc*D^b^	*Vc*K^a^	*Vc*H/H.9^c^					
2016^e^	8				8	7	1			
2017^e^	34				34	29	5			
2018	112		17		87	3		6	78	8
2019	169	1	4	2	151			150	1	11
Total	323	1	21	2	280	39	6	156	79	19

^a^Assigned based on the ‘882 assembled *V. cholerae* genomes’ dataset.

^b^Assigned based on the ‘33 mapped *Vc*D genomes’ dataset.

^c^Assigned based on the ‘456 mapped 7PET genomes’ dataset.

^d^Poor quality genome data or no coverage of the bacterial genomes; for example, in case of complete contamination by ICP1 virus genome.

^e^As reported in ref. ^7^.

**Fig. 2:**
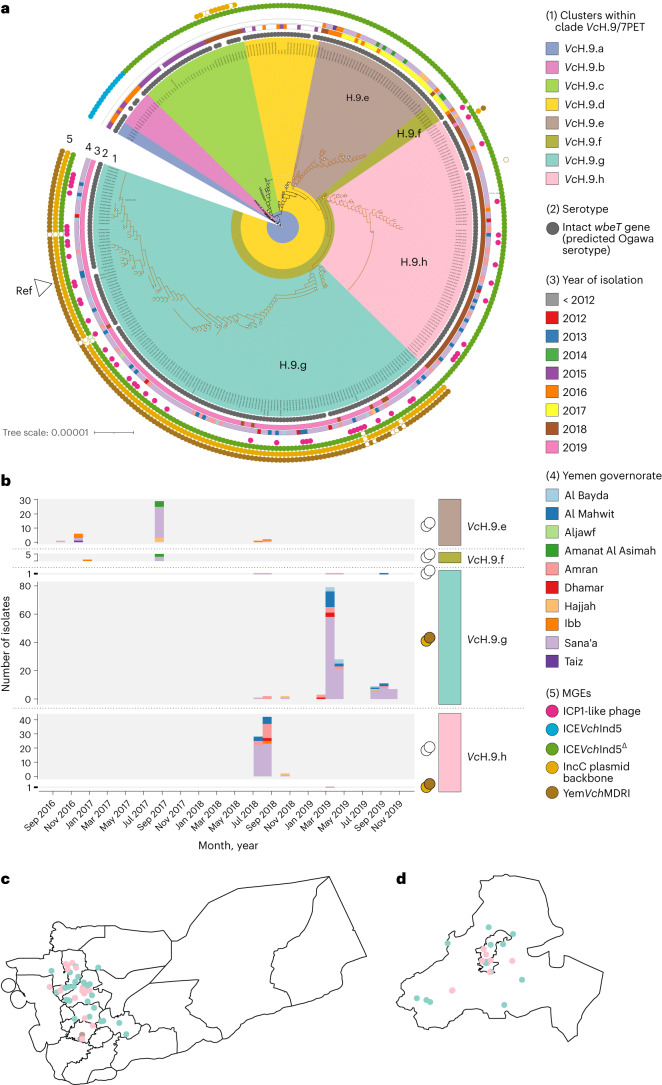
Phylogenetic diversity and spatio-temporal distribution of *V. cholerae* 7PET-T13 isolates (*Vc*H.9) from Yemen. **a**, Subtree of the ML phylogeny of 456 7PET genomes mapped to reference *Vc*H.9 strain CNRVC190243 genome, including 335/456 genomes covering *Vc*H.9 (as defined in Supplementary Fig. 5), which corresponds to the 7PET-T13 sub-lineage and close South Asian relatives. The full tree containing the 456 genomes is available as supplementary material on figshare (10.6084/m9.figshare.16595999) and was obtained based on 2,092 SNP sites from concatenated whole-chromosome alignments. Brown branches indicate the clade grouping all Yemeni 7PET-T13 isolates. Bootstrap support greater than 70% is indicated by white circles. Phylogenetic clusters within *Vc*H.9 are highlighted with background colours (legend key 1). Coded tracks outside the tree depict the serotype of isolates (ring 2) as predicted from genomic data, year of isolation when isolated in 2012 or later (ring 3) and the governorate of isolation if in Yemen (ring 4). The presence of MGEs is indicated by coloured circles in the outermost track (ring 5): ICP1-like phage (pink), SXT ICE ICE*Vch*Ind5 (blue), ICE*Vch*Ind5^Δ^ that is featuring the characteristic 10-kb deletion in the variable region III (green), IncC plasmid backbone (light brown) and the MDR PCT Yem*Vch*MDRI (dark brown); filled and unfilled circles indicate different levels of coverage in assemblies (as in Fig. 1 legend). The position of the reference sequence to which all other genomes were mapped to generate the alignment is labelled. The scale bar represents the number of nucleotide substitutions per site. **b**, Frequency of each phylogenetic subcluster among Yemen isolates per month since the onset of the Yemen outbreak. Where relevant, the cluster group is subdivided by the presence or absence of the IncC plasmid as indicated by the filled brown (present) or open (absence) circle on the right of the chart. The contribution of each governorate of isolation is indicated by the coloured portion of each bar. **c**,**d**, A map of Yemen governorates (**c**) and a focus on the Sana’a and Amanat Al Asimah governorates (inner and outer capital city; **d**), with dots corresponding to isolates, coloured by phylogenetic subcluster.

**Extended Data Fig. 5:**
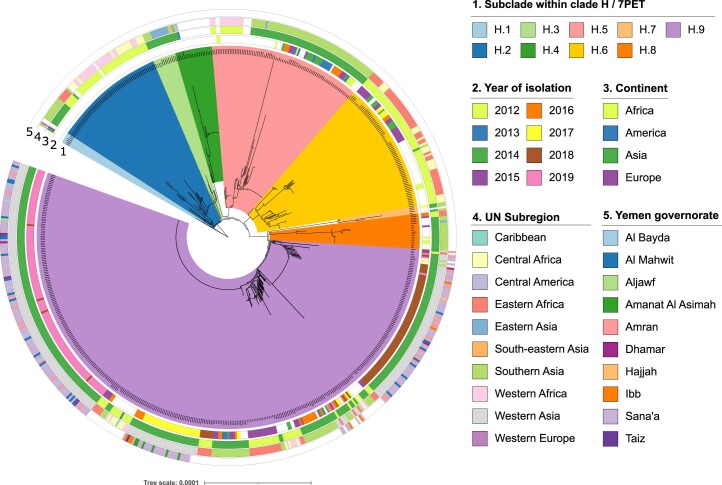
Phylogenetic diversity of *Vibrio cholerae Vc*H isolates from Yemen and contextual samples. Expanded version of phylogentic tree shown in Fig. 1, focusing on the subtree of clade *Vc*H (collapsed in Fig. 1), with details of its phylogenetic substructure.

Although highly clonal, the phylogenetic structure within the *Vc*H clade allowed it to be further subdivided into subclades *Vc*H.1 to *Vc*H.10 (Extended Data Fig. 5). All 216 *Vc*H Yemen 2016–2019 isolates fell within *Vc*H.9, which corresponds to the T13 sub-lineage of 7PET Wave 3 (ref. ^7^). We selected one representative isolate (CNRVC190243) of *Vc*H.9 and used PacBio sequencing to generate long reads in addition to the Illumina short reads already obtained, which enabled us to generate a closed hybrid assembly (Supplementary Text). To obtain greater phylogenetic resolution within *Vc*H.9, we mapped *Vc*H.9 read sets to our new *Vc*H.9 CNRVC190243 reference genome to produce a multiple whole-genome alignment. To capture minute details of the diversification of *Vc*H.9 during the outbreak, we used all 2018–2019 Yemeni isolate genomes available to us: we included seven genomes for which mapping quality was satisfactory although assembly had failed quality control, and 15 genomes for duplicate isolates independently cultured at IP (Extended Data Fig. 3), for a total of 238 *Vc*H.9 sequences. Our final alignment and resultant ‘mapped genome tree’ included another 218 previously published genomes that reside in this subclade and close outgroups, for a total of 456 genomes (Supplementary Table 6). This approach allowed us to further subdivide *Vc*H.9 into phylogenetic clusters named *Vc*H.9.a to *Vc*H.9.h. (Fig. 2a).

Yemeni 7PET *V. cholerae* genomes form a monophyletic group (clusters *Vc*H.9.e to *Vc*H.9.h; Table 1), emerging from the genetic diversity of East African genomes (clusters *Vc*H.9.c and *Vc*H.9.d), which in turn branch out of a cluster of South Asian genomes (*Vc*H.9.b), consistent with previous observations on the origins of 7PET-T13, introduced from South Asia into Africa^7,16^. Clusters *Vc*H.9.g and *Vc*H.9.h comprise the majority of 2018–2019 Yemen isolates (235/281) and form a well-supported clade (94% bootstrap) that branches from within *Vc*H.9.f.

### Spatio-temporal distribution of *V. cholerae* isolates

To delineate the evolutionary dynamics of the cholera outbreak in Yemen, we plotted *Vc*H.9 isolates by phylogenetic cluster over time (based on the date of sample collection) and between administrative divisions (linked to reporting hospital). From Fig. 2b, it is clear that each annual wave was dominated by a single cluster: 2016 and 2017 by *Vc*H.9.e, 2018 by *Vc*H.9.h and 2019 by *Vc*H.9.g. There was no evidence of geographic restriction for any of these clusters, even when accounting for dispersal over time (Fig. 2c,d, Supplementary Table 6 and online Supplementary data at 10.6084/m9.figshare.19097111). There was also no significant correlation between spatial and temporal distances, or between the spatial and phylogenetic distances (Supplementary Table 7).

However, these data did show a positive correlation between the temporal and phylogenetic distances (*R*^2^ = 0.181; Mantel test *P* < 10^−6^) (Supplementary Table 7), with root-to-tip distances significantly correlated with sampling date (Pearson’s *R*^2^ = 0.437; *P* < 10^−15^).

**Extended Data Fig. 6:**
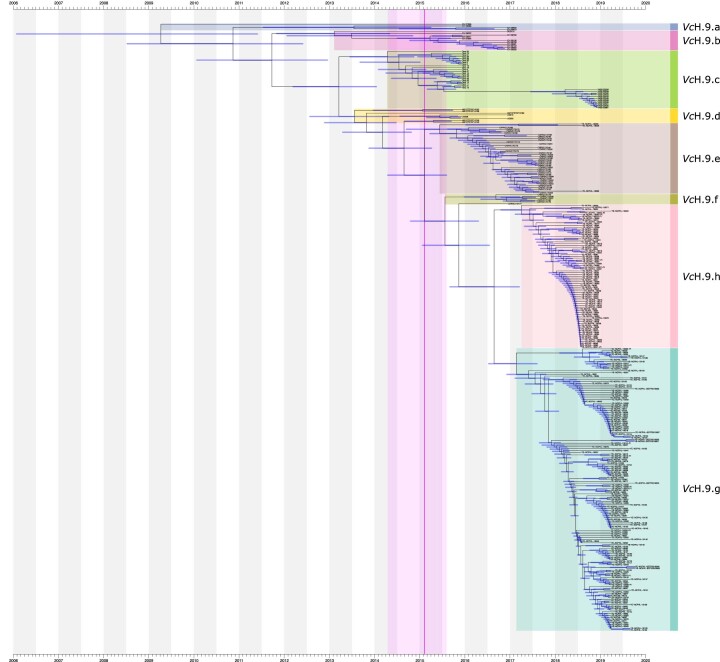
Timed phylogeny of *Vibrio cholerae Vc*H.9 isolates from Yemen and contextual samples. Timed phylogeny of 335 *Vc*H.9 genomes obtained by estimating the dates of nodes using BactDating from a recombination-free phylogeny computed with ClonalFrameML as input; the 335 genomes correspond to the *Vc*H.9 clade within the 456 mapped 7PET genome tree (as presented in Fig. 2a). Subclusters are labelled and coloured as per previous figures. X axis represents time in years. Horizontal blue bars indicate 95% confidence intervals around the node dates. The vertical purple line marks the emergence of the clade of Yemen isolates.

We inferred a recombination-free, timed phylogeny for *Vc*H.9 using a Bayesian framework (Extended Data Fig. 6), which revealed that the most recent common ancestor (MRCA) of all Yemeni *V. cholerae* 7PET-T13 genomes was estimated to have existed in February 2015 (95% confidence interval, April 2014 and July 2015). Moreover, the MRCAs for clusters *Vc*H.9.e and *Vc*H.9.f (mostly sampled in 2016 and 2017) were dated May and June 2015 respectively, and the MRCAs for clusters *Vc*H.9.g and *Vc*H.9.h (sampled in 2018 and 2019) were dated February and March 2017 respectively. In addition, we dated the MRCA of the clade grouping clusters *Vc*H.9.g and *Vc*H.9.h, which represent the majority of 2018–2019 Yemen isolates, to September 2016 (Extended Data Fig. 6).

In contrast to 7PET isolates, the distribution of non-7PET isolates (clades *Vc*D, *Vc*K and *Vpc*) across Yemen was mostly sporadic. Although we found some of the non-7PET isolates were closely related and occurred in close spatio-temporal range (Supplementary Table 5 and Supplementary data at 10.6084/m9.figshare.19097111), we found no evidence of long-range spread of the non-7PET isolates across Yemen (Supplementary Text), a pattern that thus remains characteristic of 7PET *V. cholerae* isolates linked to epidemic disease.

### Predicted phenotypic properties of *V. cholerae* isolates

Consistent with our previous report^7^, Yemeni *Vc*H.9 isolates—which all belong to 7PET-T13 sub-lineage—all carried genes or mutations known to confer resistance to trimethoprim (*dfrA1*), to nalidixic acid (*gyrA*_S83I and *parC*_S85L) and to nitrofurans (*nfsA*_R169C and *nfsB*_Q5Stop). They also carried the *Vibrio* pathogenicity island 1 (VPI-1)—encoding the toxin co-regulated pilus—and VPI-2, the *Vibrio* seventh pandemic islands I and II (VSP-I and VSP-II), and the CTX prophage, which all featured the cholera toxin genes, *ctxAB*, of the allelic type *ctxB7*. None of the non-7PET genomes from Yemen possessed a CTX phage or the *ctxAB* genes.

**Extended Data Fig. 7:**
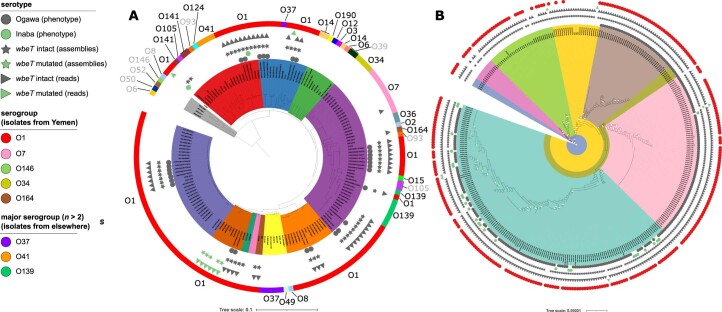
*In-silico* prediction of the O-antigen diversity among *Vibrio cholerae* from Yemen and contextual samples. Prediction of lipopolysaccharide (LPS) O-antigen serogroup and O1 serotypes projected on the isolate trees corresponding to **(A)** the core-genome tree as presented in Fig. 1a,b the subtree of the mapped 7PET genome tree as presented in Fig. 2a. Serogroup prediction are based on a best normalized BlastN score in a search against the reference LPS O-antigen biosynthetic cluster sequence from Murase et al. 2022; predictions where the best hit had a normalized percent nucleotide identity below 98% are indicated in grey font.

All Yemeni 2018–2019 *Vc*H isolates were predicted to be the O1 serogroup (except for three isolates for which genomic data were insufficient) (Supplementary Text and Extended Data Fig. 7) and were predicted to be Ogawa serotype, except two that showed a disruption in *wbeT*, indicative of an Inaba phenotype (YE-NCPHL-18053 and YE-NCPHL-19014, with gene truncation and point mutation respectively) (Supplementary Table 6).

### Genome variation of *Vc*H.9 (7PET-T13) isolates in Yemen

Given the change in antimicrobial susceptibility seen in the 2018–2019 Yemen isolates, we compared in detail the *Vc*H.9 isolate genomes from Yemen with each other and with related isolates taken elsewhere. We identified 3, 4 and 21 fixed SNPs in the crown clade containing *Vc*H.9.e,f,g,h, the clade containing *Vc*H.9.g,h, and *Vc*H.9.h, respectively (including 2, 2 and 11 non-synonymous SNPs, respectively) (Supplementary Table 8). Changes fell largely within genes predicted to be involved in carbohydrate metabolism, signal transduction and chemotaxis, none of which could be directly linked to change in virulence (Supplementary Table 8).

Previously, the 2016–2017 Yemeni isolates carried an SXT ICE differing by only three or four SNPs from the ICE*Vch*Ind5/ICE*Vch*Ban5 reference sequence (GenBank accession GQ463142.1)^17^, but which possessed a 10 kb deletion in variable region III, explaining the phenotypic loss of resistance to streptomycin, chloramphenicol and sulphonamides (only retaining resistance to trimethoprim via the *dfrA1* gene)^7^. All 2018–2019 *Vc*H.9 genomes carried the same SXT ICE deletion variant (Supplementary Table 6), showing a maximum of two pairwise SNP differences and indicating the change in AMR profile was not linked to variation in SXT ICE.

**Fig. 3:**
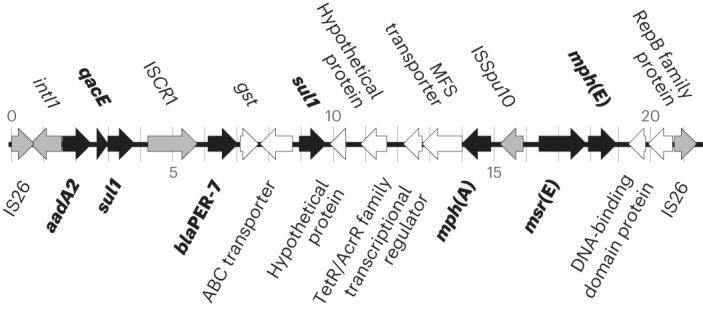
Genetic organization of the MDR PCT Yem*Vch*MDRI. AMR genes are filled in black and labelled in bold; genes encoding endonucleases transposases and other genes involved in genetic mobility are filled in grey. Genomic position is indicated by tick marks every kilobase, in reference to the pCNRVC190243 plasmid coordinates.

Looking across all genes within the pangenome, the only variation directly associated with the Yemen 2018–2019 genomes, compared with those sequenced in the period 2016–2017, was the presence of a 139 kb plasmid, which we named pCNRVC190243 (Supplementary Table 9). The backbone of this plasmid includes a replicon of the IncC type (previously known as IncA/C_2_ subtype^12^), as well as genes encoding a complete type F conjugative apparatus and a mobility region of the family MOB_H_, suggesting it is self-transmissible. Plasmid pCNRVC190243 also carries a 20 kb genomic region (which we denoted Yem*Vch*MDRI); this is a pseudo-compound transposon (PCT)—a structure bounded by IS*26* elements^18^—and includes a class 1 integron with *aadA2* encoding resistance to streptomycin and spectinomycin as a gene cassette, associated with an IS*CR*1 element carrying the extended spectrum beta-lactamase *bla*_PER-7_ gene, a structure similar to one previously seen in *Acinetobacter baumanii*^19,20^. It is also predicted to encode a quaternary ammonium compound efflux pump (*qac*), sulphonamide resistance (*sul1*) and macrolide resistance (*mph*(A), *mph*(E) and *msr*(E)) (Fig. 3). We found that pCNRVC190243 was present in 6/89 (6.7%) Yemeni *Vc*H.9 isolates from 2018, but this rose to 100% (151/151) in 2019 (Fig. 2b). This was linked to a specific phylogenetic cluster: only 1/79 (1.3%) *Vc*H.9.h isolates harboured the plasmid, compared with all (156/156) *Vc*H.9.g isolates (Fig. 2a).

### Distribution and relatedness of MDR MGEs

Analysis of the broader phylogenetic context of pCNRVC190243 and associated Yem*Vch*MDRI showed that the plasmid was also present in three *Vc*D (ST1499 and ST1020) and two *Vc*K (ST170) isolates collected in 2019 in Yemen. Comparing the full-length sequence of all pCNRVC190243 plasmids from *Vc*H.9, *Vc*K and *Vc*D isolates showed that all sequences were identical except for two isolates: one varied by a single SNP resulting in an amino acid change S71F in the sulphonamide resistance protein Sul1 (YE-NCPHL-19105; G26720A SNP); the other by a single intergenic SNP.

**Extended Data Fig. 8:**
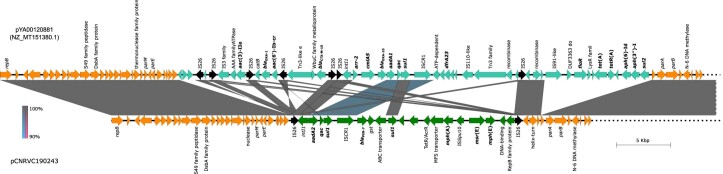
Comparison of IncC plasmids pCNRVC190243 and pYA00120881. BlastN alignment of the differing regions of IncC plasmids pCNRVC190243 and pYA00120881. Regions of similarity are highlighted by interleaving bands; band colour indicate similarity intensity and orientation of the alignment (blue, direct; pink, reverse).

We also found the Yem*Vch*MDRI element integrated into chromosome 2, without the pCNRVC190243 backbone, in all 18 of the ST555 isolates (Fig. 1 and Supplementary Text). More broadly, by searching the public genome databases (Supplementary Table 10), we found related but non-identical elements in other *V. cholerae*: an IncC plasmid, named pYA00120881 (GenBank accession MT151380), was identified in 13 closely related *Vc*H.9.a and *Vc*H.9.c isolates (Fig. 2a) that were collected in 2018 in Zimbabwe^16^. The backbones of pCNRVC190243 and pYA00120881 share 99.98% nucleotide sequence identity, but pYA00120881 carries a different MDR genomic region—featuring a *bla* gene encoding a CTX-M-15 extended spectrum beta-lactamase—inserted at the same locus (Extended Data Fig. 8). Furthermore, 59 *V. cholerae* O139 (ST69) isolates collected in China from 1998 to 2009 (publicly available genomic data released in BioProject PRJNA303115)^21,22^ carry IncC-type plasmids that show similarity to pCNRVC190243 and also include Yem*Vch*MDRI-like PCT elements, albeit lacking IS*CR*1 and the *bla*_PER-7_ gene.

**Extended Data Fig. 9:**
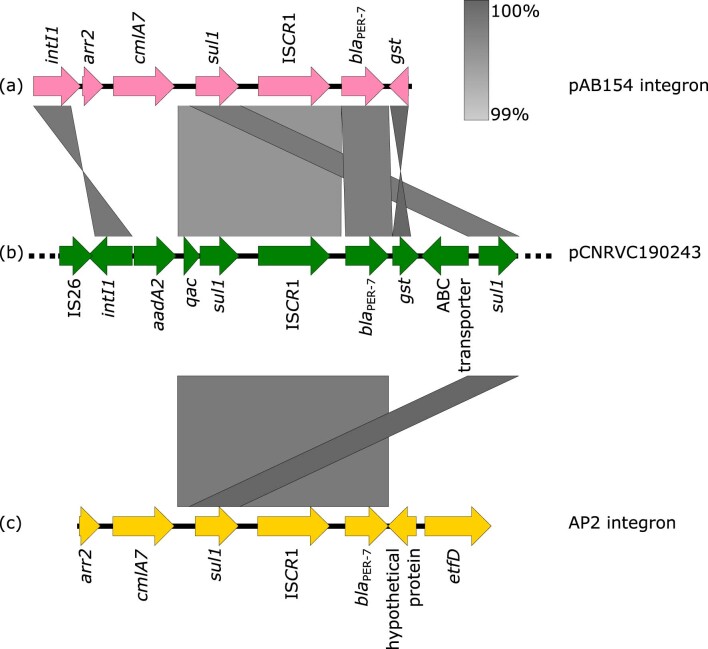
Comparison of IS*CR*1 elements. BlastN alignments of IS*CR*1 regions of (**a**) *A. baumanii* str. AB154 plasmid pAB154 integron (JQ639792.1), (**b**) pCNRVC190243/Yem*Vch*MDRI and (**c**) *A. baumanii* str. AP2 integron (HQ713678.1).

Importantly, when using the Yem*Vch*MDRI sequence alone to search the database (Supplementary Table 11), we found that the genome of *V. cholerae* ST555 strain 338360 (Supplementary Table 5) shared 100% nucleotide identity with the Yem*Vch*MDRI carried by Yemeni ST555 genomes, including the *bla*_PER-7_-carrying IS*CR*1 (IS*CR*1_*bla*PER-7_) (Supplementary Table 11). Likewise, IS*CR*1_*bla*PER-7_ has also been observed previously in the genomes of *A. baumanii* strains^19,23^ from France and the United Arab Emirates. Those from United Arab Emirates were located on the plasmid pAB154, where the sequence homology with IS*CR*1_*bla*PER-7_ extended beyond the canonical element and included Yem*Vch*MDRI flanking regions, suggesting that the IS*CR*1_*bla*PER-7_ carried by pAB154 is derived from Yem*Vch*MDRI, or a closely related element (Extended Data Fig. 9). What is more, outside *V. cholerae*, pCNRVC190243- and/or Yem*Vch*MDRI-like elements are widely distributed across other genera, with *Escherichia coli*, *Salmonella enterica* and *Klebsiella pneumoniae* genomes presenting >95% shared nucleotide *k*-mers (Supplementary Tables 10 and 11), with the latter possessing the closest matches outside *V. cholerae*. This indicates that similar regions may be widely distributed in MGEs across bacterial taxa and stably maintained in 7PET genomes.

## Discussion

In Yemen, pregnant women and children with cholera (one-third of cholera patients were aged 15 or under)^11^ were treated with erythromycin and azithromycin from 2016 until late 2018, at which point there was a sudden change in the observed antimicrobial susceptibility profile in *V. cholerae* isolated from patients. Although strains isolated in 2016–2018 were largely sensitive to antibiotics usually used for cholera treatment (excepting quinolones), by 2019 most isolates were phenotypically resistant to multiple therapeutically relevant drugs, including third-generation cephalosporins and macrolides (including azithromycin). Tetracyclines remained a viable treatment option.

Through our genomic epidemiology analysis we showed that despite significant seasonal fluctuation in incidence, the vast majority of cholera in Yemen was caused by the globally circulating 7PET-T13 lineage (*Vc*H.9) derived from a single introduction. From the inferred phylogeny we were able to subtype Yemeni 7PET-T13 genomes into four different phylogenetic clusters that dominated at different points in time during the outbreak. We observed two large clonal expansions for the sister clades that dominated in 2018 and 2019, both of which first emerged in early 2017. Our data showed that the switch in AMR phenotype coincided with the appearance in late 2018 of plasmid pCNRVC190243 in isolates belonging to the 2019 *Vc*H.9.g phylogenetic cluster. Plasmid pCNRVC190243 carries the PCT Yem*Vch*MDRI, which in turn comprises a type 1 integron and the IS*CR*1_*bla*PER-7_ element. Yem*Vch*MDRI confers resistance to third-generation cephalosporins, streptomycin (and spectinomycin), macrolides and sulphonamides, plus disinfectant tolerance provided by the *qac* gene^24^. Importantly, pCNRVC190243 (carrying the PCT Yem*Vch*MDRI) was also found in a small number of different non-7PET isolates from Yemen collected in 2018–2019 as well as the only *Vc*H.9.h isolate taken in 2019.

Although the plasmid pCNRVC190243 carrying the PCT Yem*Vch*MDRI appears to be a novel composite element, plasmid pYAM00120881, identified in *Vc*H.9 *V. cholerae* isolates from Zimbabwe in 2018^16^, shares an almost identical plasmid backbone, albeit one lacking PCT Yem*Vch*MDRI. Conversely, although rare in the public databases, elements highly similar to Yem*Vch*MDRI occurred in diverse lineages including: *V. cholerae* O139, *V. cholerae* ST555 and two *A. baumanii* strains. Detailed comparison of these PCT-related elements suggests they all are derived from a common ancestral element, and the presence of PCT Yem*Vch*MDRI in multiple ST555 strains isolated from different geographical origins suggests this element is widely distributed and has been acquired multiple times by this and other *V. cholerae* sequence types, and other bacterial genera. This is also consistent with the fact that strain 338360—a ST555 isolate from India—is distinguished from our Yemeni ST555 reference strain CNRVC019247 by 436 SNPs over both chromosomes, indicating that the Yemen isolates, although related and carrying the same PCT, do not share a recent common ancestor with strain 338360. Furthermore, because pCNRVC190243 and Yem*Vch*MDRI can self-mobilize, it is possible that Yem*Vch*MDRI would have transposed onto the progenitor of pCNRVC190243 in the context of the Yemen cholera outbreak, and therefore be subject to antibiotic selection. However, more data would be needed to confirm this.

What is clear is that acquisition of the pCNRVC190243 plasmid containing the Yem*Vch*MDRI element by an ancestor of the Yemeni *Vc*H.9.g isolates was followed by its dramatic spread, a clonal expansion that we show occurred in 2018, a time when the treatment regimen was to treat symptomatic cases with macrolides. It is possible to explain the distribution of pCNRVC190243 by multiple acquisitions of the plasmid from independent sources or, more parsimoniously, as direct horizontal gene transfer events between the epidemic and endemic *V. cholerae* strains. The large population sizes attained by the epidemic lineages in Yemen make spillover from the dominant cluster at the time, *Vc*H.9.g, probable. Consistent with this, our microbiology, serotyping and sequencing data showed that four of the samples analysed here contained both ST555 and 7PET strains (Supplementary Text), indicative of a limited number of mixed infections from individual patients.

Recently, it has been shown that the presence of two defence systems, called DdmABC and DdmDE, destabilizes plasmids in *V. cholerae* 7PET lineage isolates. However, large IncC-type plasmids are an exception because the Ddm systems only give a competitive disadvantage to plasmid-bearing cells, which is probably overcome in the presence of selection for the function of the plasmid cargo; for example, antibiotic resistance^25^. Having said that, although other MDR IncC plasmids have been previously observed in *V. cholerae* in Democratic Republic of the Congo, Kenya and Zimbabwe, unlike in Yemen these were linked only to sporadic cases or small-scale cholera outbreaks, despite uncontrolled use of antibiotics, and were not linked to T13 lineage isolates. Apart from the Yemen cholera outbreak, the only other example of a massive clonal expansion of a *V. cholerae* lineage carrying an MDR IncC plasmid was the T13 *Vc*H.9.c clone responsible for the Zimbabwean cholera outbreak of 2018, which lasted six months with over 10,000 suspected cases. This observation was so striking that through detailed comparative analysis of the genomes we showed that T13 isolates uniquely carry a 10 kb deletion in the SXT ICE (ICE*Vch*Ind5/ICE*Vch*Ban5). Because the presence of SXT ICE has been proposed previously to prevent the stable replication of IncC-type plasmids through an unknown functional interference^7,16^, it is possible this deletion impairs the putative interference mechanism and allows an SXT ICE and an IncC plasmid to stably propagate together in T13 strains.

The emergence of this MDR pathogen demonstrates the necessity of continued genomic surveillance of the microbial population associated with the ongoing Yemen cholera outbreak, and for new outbreaks that may take place in regionally connected areas. Such surveillance will enable Yemeni public health authorities to rapidly adapt clinical practices to minimize AMR selective pressures. This also warrants increased efforts in research on the molecular mechanisms and evolution of interactions between MGEs, to learn about the constraints ruling their colonization of bacterial genomes. Such knowledge is essential for us to be able to disentangle the role of MGEs from that of their bacterial hosts in driving epidemics, so to propose practical definitions of pathogens that focus on the relevant genes, mobile elements or prokaryotic organisms, and to implement appropriate molecular epidemiology surveillance schemes.

## Methods

### Definitions and surveillance data

Cholera cases were notified to the MPHP and recoded through the Electronic Disease Early Warning System^2^. Suspected and confirmed cholera cases were defined according to the World Health Organization in a declared outbreak setting. Briefly, a suspected case is any person presenting with or dying from acute watery diarrhoea and a confirmed case is a suspected case with *V. cholerae* O1 or O139 infection confirmed by culture.

### Sample and metadata collection, and microbiological testing

Clinical samples, that is stool and rectal swabs, were collected in Yemen by epidemiological surveillance teams from suspected cholera cases during 2018 and 2019^11^, and were transported to the NCPHL in the capital city Sana’a in Cary–Blair transport medium (Oxoid). To probe the diversity of vibrios shed by unreported cholera cases, as well as *V. cholerae* that may naturally occur in effluent waters, environmental samples were collected during the day time in October 2019 from the sewage system in and around Sana’a city and then transported to NCPHL for testing; each sample was collected in sterile bottles containing enrichment media comprised of 250 ml of sewage and alkaline peptone broth (Difco Laboratories) at a 1:1 ratio and incubated for 20 h at room temperature, including the transportation time to the NCPHL, and processed as described previously^26^. All samples were cultured and identified according to Centers for Disease Control and Prevention guidelines^27^. Resistance to antibiotics was tested by the disk diffusion method according to the Clinical & Laboratory Standards Institute guidelines^28^ for a range of antibiotics as described in Supplementary Table 2.

Live clinical isolates (*n* = 120) were sent to IP, where only 21 samples were culture positive because of poor sample preservation during shipment (Supplementary Table 3 and Extended Data Fig. 3), leading to the final isolation of 22 *V. cholerae* strains (including two from mixed culture YE-NCPHL-18020). Strains re-isolated at IP were characterized by biochemical and serotyping methods according to standard practice of the French National Reference Centre for Vibrios and Cholera (CNRVC)^29^. Separate antibiotic susceptibility testing (Supplementary Tables 2 and 3) was performed by the disk diffusion method according to EUCAST guidelines^30^ and minimum inhibitory concentration determination using the Sensititre (Thermo Fisher Scientific) and Etest (bioMérieux) systems. Interpretation into S (susceptible), I (intermediate) and R (resistant) categories was performed according to the 2020 edition of EUCAST recommendation on interpretation of the diameter of the zones of inhibition of *Enterobacteriaceae*^31^, and to the 2013 Comité de l’Antibiogramme de la Société Française de Microbiologie standards for *Enterobacteriaceae*^32^ for antibiotics for which critical diameters are no longer reported in the latest published guidelines. *E. coli* CIP 76.24 (ATCC 25922) was used as a reference strain.

### DNA extraction and sequencing

Genomic DNA was extracted at the NCPHL from subcultures inoculated with single bacterial colonies and grown in nutrient agar (Oxoid) at 37 °C overnight according to the manufacturer’s instructions (Wizard Genomic DNA Purification kit, Promega). Genomic DNA samples (derived from 10 environmental and 250 clinical samples, which includes the 120 samples sent to IP) were sent to the Wellcome Sanger Institute (WSI) and sequenced on the WSI sequencing pipeline (Extended Data Fig. 3) using the Illumina HiSeq platform X10 as described previously^33^.

### Genome assembly and annotation

The 260 sequencing read sets produced at the WSI (Extended Data Fig. 3) were processed with the WSI Pathogen Informatics pipeline^34^; quality of sequencing runs was assessed based on quality of mapping of 10% reads to the genome of reference strain N16961 (GenBank Assembly accession GCA_900205735.1) using the Burrows–Wheeler Aligner^35^; read sets passed the check if at least 80% bases were mapped after clipping, the base and indel error rate were smaller than 0.02, and fewer than 80% of the insert sizes fell within 25% of the most frequent size. Contamination was assessed manually based on Kraken classification of reads using the standard WSI Pathogen reference database, which contains all viral, archaeal and bacterial genomes and the mouse and human reference published in the RefSeq database as of 21 May 2015 (Supplementary Table 4). Sequences were assembled de novo into contigs as described previously^36^, using SPAdes v.3.10.0 as the core assembler^37^. Poor assemblies were filtered out if differing by more than 20% from the expected genome size of 4.2 Mb, or when more than 10% of reads were assigned by Kraken to an organism other than *V. cholerae* (notably including the *Vibrio* phage ICP1) or to synthetic constructs, or were unclassified. This led to the exclusion of 28 genome assemblies, resulting in 232 high-quality assembled genomes. The genomes of strains CNRVC190243 and CNRVC190247 were assembled based on long and short reads using a hybrid approach with UniCycler^38^ v.0.4.7 and v.0.4.8, respectively, using pilon^39^ v.1.23 for the polishing step, to produce high-quality reference sequences comprised of both chromosomes and, for strain CNRVC190243, of an additional plasmid, pCNRVC190243. New genomes were annotated with Prokka (v.1.5.0)^40^.

### Contextual genomic data

To provide phylogenetic context, we also included in this analysis previously published genome sequences from a globally representative set of isolates (‘assembled *V. cholerae* genomes’ dataset; *n* = 882). We first gathered genome assemblies generated at the WSI using the pipeline described above based on previously published short reads sets from *V. cholerae* isolates belonging to sub-lineage T13 of 7PET Wave 3 (7PET-T13) and from strains isolated in the close spatio-temporal context that is within a decade in Africa and South Asia (where the ancestor of T13 is thought to originate^7^). These include all 42 Yemen 2016–2017 isolates^7^, 103 recent isolates from East Africa including from Kenya^7^, Tanzania^41^, Uganda^42^ and Zimbabwe^16^, and 74 isolates from South Asia^43^. In addition, we included genomes spanning the wider diversity of *V. cholerae*, including all 119 genomes from China^44^, as well as 312 genomes from the collections of contextual genomes used in our previous studies^7,33^. Together with the 232 Yemen 2018–2019 isolate genome assemblies (see above), our final dataset consisted of 882 assembled *V. cholerae* genomes (Supplementary Table 5 and Extended Data Fig. 3).

### Characterization of genomic features of interest

We first predicted the presence of various genomic features by searching genomes against reference databases. The presence of AMR genes, plasmid replicon regions or virulence factors were predicted using ABRicate^45^, searching the reference databases NCBI AMR+^46^, Plasmidfinder^47^ or VFDB^48^, respectively. We searched for the presence of conjugation apparatus (as a sign of plasmids or ICEs) and CRISPR–Cas arrays and subtyped these systems using MacSyFinder (v.2.1)^49^ with models CONJScan (v.2.0.1) and CasFinder (v.3.1.0). Genomes positive for Cas systems were further analysed with CRISPRCasFinder^50^ on the Pasteur Institute Galaxy server to retrieve CRISPR arrays.

In addition, we searched for sequences with significant similarity to previously described mobile elements relevant to our study. BLASTN^51^ (v.2.7.1+, with default parameters) was used to identify known MGEs: the SXT ICE ICE*Vch*Ind5 (GenBank accession GQ463142.1); ICP1-like vibriophages ICP1_VMJ710 and ICP1_2012_A (GenBank accessions MN402506.2 and MH310936.1, respectively)^52^ and the ICP1-like *Vibrio* phage YE-NCPHL-19021, which genome was the only assembled contig from the reads obtained from sample YE-NCPHL-19021 (this study; GenBank accession MW911613.1); the IncC-type plasmid pCNRVC190243, obtained from the hybrid assembly of strain CNRVC190243 described above (this study; ENA sequence accession OW443149.1); the MDR PCT Yem*Vch*MDRI, extracted from this plasmid (positions 16,442 to 36,862); PICI-like elements (PLE) 1, 2 and 3 (GenBank accessions KC152960.1, KC152961.1, MF176135.1)^53,54^. Absence of elements was verified at the read level as described below. Sequences similar to the reference sequences of the plasmid pCNRVC190243, the MDR PCT Yem*Vch*MDRI and the ICP1-like phage genome YE-NCPHL-19021 were also searched in a database of 661,405 genome assemblies^55^ using a *k*-mer based COBS index^56^; alignment of best matches were further characterized using BLASTN.

### In silico classification of *V. cholerae*

To predict the antigenic serogroup, we used BLASTN to screen the assemblies against a reference database of sequences of lipopolysaccharide O-antigen biosynthetic gene clusters that were delineated in the genome of reference strains for each known serogroup^57^. Best reference locus matches were identified as those with the highest combined score, summing scores of all local alignments, except when multiple local alignment overlapped in which case only best-scoring alignments were retained^58^.

For O1 serotype prediction (Inaba or Ogawa), we used a combination of approaches including BLASTN search against the 882 assembled *V. cholerae* genomes (as described above) and ARIBA^59^ v.2.14.6+ (with default parameters) to screen the sequencing read sets against the *wbeT* gene sequence from strain NCTC 9420 (positions 311,049–311,909 of GenBank accession CP013319.1) as a reference, as described previously^33^. Multilocus sequence typing of non-7PET isolates was conducted on PubMLST.org^60^ under the non-O1/non-O139 *V. cholerae* seven-gene typing scheme.

### Identification of single nucleotide variants

For variant calling, Illumina short reads from 7PET and *Vc*D genomes were mapped against the reference genomes from strains CNRVC190243 and CNRVC190247, respectively (456 ‘mapped 7PET genomes’ and 33 ‘mapped *Vc*D genomes’ datasets, respectively), and all genomes were mapped against the in-house MGE database described above. We mapped all 260 short-read sets from 2018–2019 Yemeni isolates sequenced at the WSI, including those 28 read sets that assembly showed low coverage or appeared contaminated with phage genomes (Supplementary Table 4); to recover variation data evidenced at the read level, provided reads were mapped at a sufficient depth (see below). We also mapped read sets from the 21 strains sequenced at the IP, and from contextual isolates of the 7PET-T13 sub-lineage and close relatives (Contextual genomic data), for a total of 468 mapped genomes. Reads were trimmed with Trimmomatic, mapped to both CNRVC190243 reference chromosomes with BWA-MEM and the IncC plasmid pCNRVC190243. Mapped genomes with an average read depth below 5× over the two chromosomes were deemed of insufficient read depth and were excluded (12 read sets mapped to CNRVC190243, all from this study and generated at WSI, were excluded for a final set of 456 mapped 7PET genomes (Supplementary Table 5); no read set mapped to CNRVC190247 was excluded). We used the software suite samtools/bcftools^61^ v.1.9 to call single nucleotide variants with a minimum coverage of 10× read depth. Resulting consensus sequences were combined into a whole-genome alignment, which was processed with snp-sites^62^ to produce an SNP alignment.

Overall genome similarity was assessed by computing SNP distances based on the above alignments using the function ‘dist.dna’ from the R package ‘ape’ ^63^, and ANI (accounting for unaligned regions) was computed using fastANI^64^ v.1.3 with default parameters.

### Phylogenetic inference

The Pantagruel pipeline^65^ was used to infer a maximum-likelihood (ML) ‘core-genome tree’ using the ‘-S’ option and otherwise default parameters. Briefly, 291 single-copy core-genome genes were extracted from the 882 assembled *V. cholerae* genomes; these 291 markers represent a strict definition of the core genome that is those genes occurring once and once only in all genomes of the dataset. This restrictive definition was intended to retain only genes with an expected high degree of sequence conservation and relatively low prevalence of horizontal gene transfer compared with other core genes, towards a robust phylogenetic inference at the species scale. The alignments of these 291 single-copy core-genome genes were concatenated and the resulting supermatrix was reduced to its 37,170 polymorphic positions, from which a ML tree was computed from RAxML^66^ v.8.2.11 (model ASC_GTRGAMMAX using Stamatakis’ ascertainment bias correction; one starting parsimony tree; 200 rapid bootstraps for estimating branch supports). Phylogenies were also inferred from whole-genome alignments of the concatenated consensus sequences of both chromosomes from the SNP alignment of the 456 mapped 7PET genomes and 33 mapped *Vc*D genomes. These alignments contained 2,092 and 91,312 polymorphic positions, respectively, and were used as input to RAxML-NG^67^ v.1.0.1 to build the ML ‘mapped genome trees’ using the following options: ‘all --tree pars{10} --bs-trees 200 --model GTR+G4+ASC_STAM’. Alternative topologies were compared using RAxML-NG option ‘--sitelh’ to generate per-site likelihood values and the ‘SH.test’ function from the ‘phangorn’ R package^68^ to test hypotheses.

The 882 assembled *V. cholerae* core-genome tree was rooted using the clade of sequences identified as *V. paracholerae*^15^ as an outgroup. The remaining part of the tree (*V. cholerae sensu stricto*) was subdivided into clades named *Vc*A to *Vc*K based on visual examination with the aim to coincide with previously described lineages such as 7PET and Gulf Coast among others, or based on even subdivisions of the tree diversity. *Vc*H, corresponding to the 7PET lineage, was further subdivided into clades of even depth, named subclades H.1 to H.9. The 456 mapped 7PET genomes were similarly classified into clusters based on the tree topology, with genomes assigned to subclades named *Vc*H.5, *Vc*H.6, *Vc*H.8 or *Vc*H.9 (according to their position in the 882 assembled *V. cholerae* core-genome tree). Genomes belonging to *Vc*H.9, which corresponds to the 7PET-T13 sub-lineage, were further separated into *Vc*H.9.a to *Vc*H.9.h, based on visual examination of the tree structure and aiming to maximize uniformity of the spatio-temporal metadata associated to genomes in each cluster; clusters correspond to clades, either entirely or at the exclusion of another cluster included in the clade; that is, genome clusters can emerge from each other. Final trees for the mapped genome datasets were rooted manually according to the branching pattern in the 882 assembled *V. cholerae* core-genome tree, the diversity of which encompasses that of the mapped genome trees.

**Extended Data Fig. 10:**
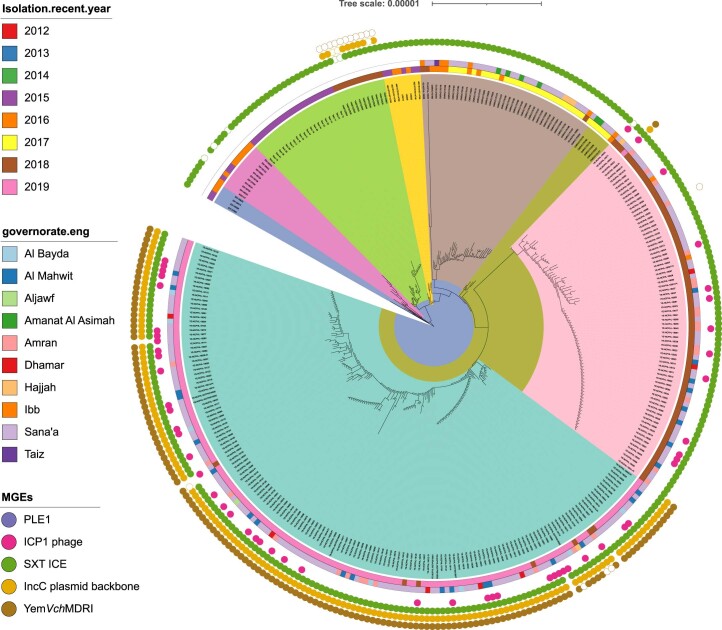
Recombination-free phylogeny of *Vibrio cholerae Vc*H.9 isolates from Yemen and contextual samples. Tree computed from the same alignment as in Fig. 2a, but using ClonalFrameML to infer a recombination-free phylogeny reflecting the clonal propagation of the organism.

From a subset of the 456 mapped 7PET genome alignments (*n* = 335) corresponding to *Vc*H.9, a recombination-free phylogeny (Extended Data Fig. 10) was inferred using ClonalFrameML^69^ v.1.11 with default parameters and using the ML mapped genome tree (restricted to the *Vc*H.9 genome tips) as a starting tree. BactDating^70^ v.1.1 was then used to estimate a timed phylogeny (using 100,000 Monte Carlo Markov chain iterations and otherwise default parameters) of the Yemen 2016–2019 genomes and relatives using the ClonalFrameML tree and day-resolved dates as input; median day of the year of isolation was used for isolates where these data were missing. Three independent chains were run from different random seeds and yielded close results.

Supporting data for phylogenetic analyses of the 882 assembled *V. cholerae*, 456 mapped 7PET genomes and 33 mapped *Vc*D genomes are available on the figshare repository (Data Availability).

### Correlation of spatio-temporal and phylogenetic distances

GPS data associated with the site of sample collection (health centres) were used to compute spatial geodetic distances using R script ‘gps_coords.r’^71,72^. Temporal distances were computed from the difference between day of collection (available only for 2018 and 2019 Yemen isolates). Phylogenetic distances were computed from the mapped genome tree using the function ‘cophenetic’ from the core R package ‘stats’^73^. Spatial, temporal and phylogenetic distances were compared using a Monte Carlo approximation of the Mantel test as implemented in the ‘mantel.randtest’ function from the R package ‘ade4’^74^, using 100,000 permutations to compute the simulated *P* value. Maps showing the distribution of genomes clusters over the Yemen territory and in the region of Sana’a were obtained using QGIS 3.16.3 and the QuickOSM API to retrieve OpenStreetMap data, specifically level 4 administrative boundaries (governorates) in Yemen (last accessed 11 February 2021).

### Pangenome analysis and clade-specific SNPs

On one hand, the synteny-aware pangenome pipeline Panaroo^75^ v.1.2.3 was run on the 882 assembled *V. cholerae* genome set with the option ‘--clean-mode strict’ and default parameters otherwise. On the other hand, a combined VCF file containing information on all SNP variation within the 456 mapped genome set was obtained using the ‘bcftools merge’ command. To identify clade-specific SNPs and accessory gene presence/absence patterns, we used custom R scripts^58^ to compare the combined VCF file and the gene presence/absence table output of Panaroo, respectively, with the mapped genome tree. Based on lists of genomes assigned to various clades and clusters (Results), we identify SNPs or accessory genes that are specific of a focus clade in contrast to a background group or a sister clade, considering the contrast significant when the Bonferroni-corrected *P* value is below 0.05 and when the frequency of an allele is above 0.8 in the focus clade and below 0.2 in the background clade, or conversely. Pangenome analysis files are available on figshare (10.6084/m9.figshare.19519105). Putative anti-phage defence systems were searched by testing correlation of presence/absence patterns between ICP1-like phage and each pangenome gene cluster; only associations with Pearson correlation coefficients lower than −0.9 or greater than 0.9 and *P* values lower than 10^−5^ were retained as significant.

### Ethics and approval of sampling

This study is based exclusively on bacterial isolates and derived genomic DNA extracts and complies with all relevant ethical regulations as follows. None of the human samples from which the strains were isolated were collected specifically for this study, as all were collected as part of the cholera outbreak surveillance effort led by the NCPHL. The metadata associated with the samples and retained in the study are age and sex, as well as place of hospitalization, which do not identify the patient and do not warrant informed consent or ethics committee approval. Bacterial isolate cultures were later sent to IP, while genomic DNA extracts were transferred to the WSI. We note that Yemen is not a signatory to the Nagoya Protocol and therefore does not require transfer authorization under international law.

### Reporting summary

Further information on research design is available in the Nature Portfolio Reporting Summary linked to this article.

### Supplementary information

Supplementary Information Supplementary Text and Figs. 1 and 2.

Reporting Summary

Supplementary Tables Supplementary Tables 1–14.

## Data Availability

Short-read genomic data sequenced at the WSI were deposited at the European Nucleotide Archive (ENA) under the BioProject PRJEB34436. Four of the resulting assemblies comprised a single 123-kb contig corresponding to the ICP1-like phage; these assemblies were deemed uncontaminated and complete ICP1-like phage genomes and were deposited to GenBank under the accessions MW911612–MW911615. Complete hybrid genome assemblies for reference strains CNRVC019243 and CNRVC019247 were deposited to the ENA under the BioProject accessions PRJEB52123 and PRJEB47951 (Assemblies GCA_937000105 and GCA_937000115), respectively. Supplementary data are available online on the Figshare repository, under the following digital object identifiers (doi): 10.6084/m9.figshare.16595999, 10.6084/m9.figshare.16611823, 10.6084/m9.figshare.18304961, 10.6084/m9.figshare.19097111, 10.6084/m9.figshare.19519105, 10.6084/m9.figshare.23653971, 10.6084/m9.figshare.23849034.

## Code availability

All custom code used in this study was made available in a git repository publicly availably on GitHub at https://github.com/flass/yemenpaper (release v.0.3; 10.5281/zenodo.8221344).
