# Supplementary material for: Genomic epidemiology reveals multidrug resistant plasmid spread between Vibrio cholerae lineages in Yemen
Source: Nat Microbiol. 2023 Sep 28;8(10):1787–98. doi: 10.1038/s41564-023-01472-1 (PMC10539172; doi:10.1038/s41564-023-01472-1)
Supplement: Supplementary file 1 — Supplementary Text and Figs. 1 and 2. [file 41564_2023_1472_MOESM1_ESM.pdf]

nature microbiology

Article

https://doi.org/10.1038/s41564-023-01472-1

Genomic epidemiology reveals multidrug resistant plasmid spread between *Vibrio cholerae* lineages in Yemen

In the format provided by the authors and unedited

<u>Supplementary Text</u>

Samples and cultures

Out of the 4,375 samples confirmed to contain *V. cholerae* at the National Centre of Public Health Laboratories in the capital city Sana'a (NCHPL; Sana'a, Yemen), 260 cultures were used to prepare genomic DNA that were sent to the Wellcome Sanger Institute (WSI; Hinxton, UK). In parallel, an overlapping set of 120 live cultures were sent to the Institut Pasteur (IP; Paris, France), where only 21 could be revived (see Extended Data Figure 3). Whilst samples received at both WSI and IP were derived from the same samples sharing the same nomenclature with the 'YE-NCHPL-' prefix, genomic and/or phenotypic information obtained from corresponding samples showed discrepancies (Table S13). Based on whole-genome phylogenies, we found that, out of 21 WSI/IP genome pairs, 10 had undistinguishable genotypes (branching as immediate relatives in the tree), and 6 branched separately on the tree but belonged to the same phylogenetic cluster – suggesting that the few observed genotypic differences could be ascribed to sequencing or mapping errors – confirming in 16 cases the genetic uniqueness of the twice-sequenced isolates. However, 4 genome pairs had completely distinct genotypes, belonging to cluster *Vc*H.9.h/7PET-T13 (all 4 genomes from strains isolated at NCHPL and sequenced at WSI) or *Vc*D/ST 555 (all 4 genomes from strains sub-cultured and sequenced at IP); the last genome sequenced at IP had no useable counterpart at WSI due to sequencing failures. This discrepancy for 4 samples may have stemmed from the presence of mixed strain cultures in the original samples resulting in different dominant clones being sequenced or subcultured in the respective recipient institutes. This hypothesis is supported by positive PCR screens for an O1 *V. cholerae*-specific marker (O1-type *rfb* operon)[1] from samples that resulted in non-O1/non-O139 strain cultures at IP but yielded O1 strain genomes at the WSI. As a result, we chose to use two separate nomenclatures for samples received at WSI and IP, with the IP sample names bearing the '-PI' suffix (Table S3), whereas the WSI samples retained the original name.

Genomic DNA preparation for long-read sequencing

Two MDR *V. cholerae* strains were selected among the 22 held at the IP for long-read sequencing. The first strain, CNRVC190243 (YE-NCPHL-19014-PI), a 7PET *V. cholerae* O1 strain was sequenced by Single-Molecule Real-Time (SMRT) sequencing (Pacific Bioscience). The genomic DNA was prepared at the IP as follows: strain CNRVC190243 was cultured in Brain-Heart-Infusion (BHI) broth (Difco) overnight at 37 °C with shaking (200 rpm—Thermo Scientific MaxQ 6800). Then, 100 µL of the overnight culture was inoculated into a 10 ml BHI broth and cultured for 2 hours at 37°C with shaking. After centrifugation, the bacterial cells were processed as described previously[2], except that MaXtract High Density columns (Qiagen) were used (instead of phase lock tubes) and DNA was resuspended in molecular biology grade water (instead of 10 mM Tris pH 8.0). Library preparation and the sequencing were performed by the GATC platform (Eurofins Genomics Europe Sequencing GmbH; Konstanz, Germany) using their standard genomic library protocol and PacBio RS sequencer. The second strain, CNRVC190247 (YE-NCPHL-18035-PI), a non-O1/non-O139 *V. cholerae* strain further

characterized as ST555, was sequenced using the MinION nanopore sequencer (Oxford Nanopore Technologies). Genomic DNA was prepared at the IP as follows: strain CNRVC19247 was cultured in alkaline nutrient agar (casein meat peptone E2 from Organotechnie, 20 g; sodium chloride from Sigma, 5 g; Bacto agar from Difco, 15g; distilled water to 1 L; adjusted to pH 8.4; autoclavated at 121°C for 15 min) overnight at 37°C. A few isolated colonies of the overnight culture were inoculated into a 20 ml of Brain-Heart-Infusion (BHI) broth and cultured until a final OD_{600}=0.8 at 37°C with shaking (200 rpm). After centrifugation, the bacterial cells were processed as described above. The library was prepared according to the instructions of the "Native barcoding genomic DNA (with EXP-NBD104, EXP-NBD114, and SQK-LSK109)" procedure provided by Oxford Nanopore Technology. The sequencing was then completed using the MinION Mk1C apparatus (Oxford Nanopore Technologies). The genomes of 21/22 strains cultivated at the IP (all but CNRVC190251, which was isolated later; Table S3) were also sequenced using Illumina short-read technology at the IP using the equipment and services of the iGenSeq platform at the Institut du cerveau et de la moëlle épinière (Paris, France) from genomic DNA extracted with the Maxwell 16-cell DNA purification kit (Promega) in accordance with the manufacturer's recommendations.

Spatiotemporal distribution of non-7PET *V. cholerae* isolates

The *V. paracholerae* and *Vc*D (ST1499) isolates obtained from Yemen had no close phylogenetic relatives taken from Yemen in this study. Contrastingly, the ST555 *Vc*D genomes formed a cluster of 18 closely-related isolates – ten of which have high-quality genome assemblies (Figure 1 and Table S4) – which differed from each other by 0 to 10 SNPs (average 99.98% ANI similarity). Of these 18 isolates, 13 were isolated over a period of 11 days in late July/early August 2018, two at the end of August, two in October 2018, and one in March 2019 (Table S6). They were obtained from patients in the neighbouring governorates of Sana'a (n=7), Al Mahwit (n=4) and Amran (n=1), which are surrounding the capital city. These are likely explained by repeated acquisitions from a point source, although we cannot rule out that they stem from a small-scale outbreak, as has been reported previously for non-7PET strains[3,4]. Genomes from other known ST555 isolates, including strains reported as linked to travelers returning from India in September 2015 and July 2016 (strains 229152 and 338360)[5], as well as closest relatives from our core-genome tree, were gathered to build a tree of mapped *Vc*D genomes using the 2018 Yemen strain CNRVC190247 complete genome as a reference (Additional Supplementary Figure 1, Table S13). The closest relative to Yemeni ST555 isolates, isolate 338360, differs from the *Vc*D ST555 genomes sequenced here by between 763-800 SNPs, ruling out direct clonal relationships.

Other instances of closely related isolates (ANI > 99.97%) from non-7PET clades (Table S14) were found, always in very localised areas: a pair of *Vc*K isolates (ST170) were sampled the same week of March 2019 from patients in Sana'a governorate, while a pair of *Vc*D isolates (ST1020) were taken on consecutive days in October 2019 from sewerage in Sana'a (Figure 1; Table S4).

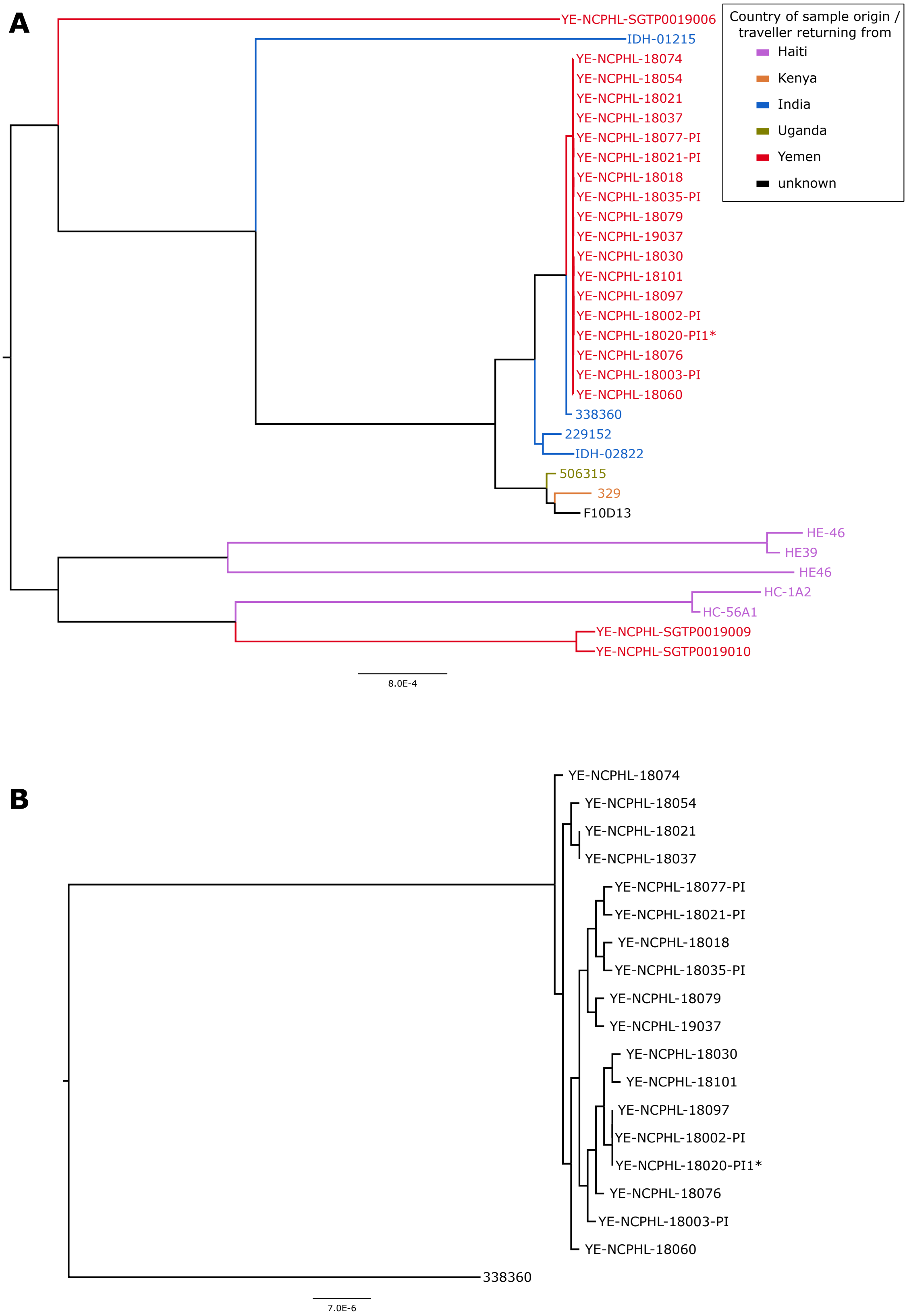

Additional Supplementary Figure 1: Phylogenetic diversity of *Vibrio cholerae* *Vc*D/ST555 isolates from Yemen and contextual samples

A. Maximum-likelihood phylogeny of the 33 mapped *Vc*D genomes. The tree was obtained based on the 91,312 SNP sites from concatenated whole-chromosome alignments. **B.** Subtree of the tree in A, focusing on the ST555 isolates from Yemen, and their closest relative, strain 338360.

In-silico serotyping of 7PET isolates

Of the 216 Yemeni 2018-2019 *Vc*H/7PET isolates, 213 were predicted to produce a serogroup O1 LPS O-antigen based on presence of a full biosynthetic gene cluster; in the three remaining assemblies this genomic region was interrupted (YE-NCPHL-19012) or completely missing (YE-NCPHL-18033 and YE-NCPHL-19140), likely due to limited genome sequence coverage (Table S4). These predictions, along with serotype predictions (see main text) were imperfectly reflected by the results of serological assays conducted at NCPHL (Table S2; Extended Data Figure 7), suggesting issues in initial laboratory testing (see next section).

In-silico phenotyping of non-7PET isolates

None of the non-7PET genomes from Yemen possessed a CTX phage or the *ctxAB* genes. However, isolates belonging to *Vc*K (ST170), which were derived from the stool of patients presenting cholera-like disease, carried all the genes coding for the TCP. These *ctxAB*$^-$, *tcpA*$^+$ *Vc*K genomes also carried the O1 LPS O-antigen biosynthetic gene cluster, consistent with what has been seen previously in related non-7PET isolates[6]. The genomes of the *Vc*D isolates belonging to ST555, ST1020 and ST1498 and the *V. paracholerae* isolate (ST1499), carried LPS O-antigen biosynthetic gene clusters that were most similar to those known to encode the serogroups O7, O34, O164 and O146, respectively[7]; these were conserved within, and specific to, each ST (Table S5; Extended Data Figure 7).

Comparison of phenotypic data obtained from experimenal characterisation and *in silico* predictions

Phenotypic characterisation of *V. cholerae* isolates at NCHPL included serotyping: testing with antisera for O1 serogroup serotypes Inaba and Ogawa. The isolates corresponding to the 260 Illumina-sequenced genomes reported in this study were all positive for either Ogawa (n=230) or Inaba (n=30) (Table S2). However, these results proved inconsistent with our *in silico* serotyping using ARIBA (see Methods), showing that only 218/241 testable genomes were of serogroup O1, and among them, only two were predicted to be of the Inaba serotype (isolates YE-NCPHL-18053 and YE-NCPHL-19014 = YE-NCPHL-19014-PI = CNRVC190243) (Tables S2, S4). Serotyping conducted on the subset of the samples sub-cultured at the IP (Figure S3) showed a higher degree of consistency with the *in silico* serotype predictions, with only strain YE-NCPHL-19015-PI serotyped as Inaba while *in silico* serotyping of corresponding genome assembly for isolate YE-NCPHL-19015 predicted an Ogawa serotype (Table S2).

In addition, antibiotic sensitivity testing at NCHPL showed an ampicillin resistant phenotype for all tested strains, including those not carrying the pCNRVC190243 plasmid or without any other potential resistance determinant as predicted using Abricate (see Methods; Table S2); and ampicillin sensitivity was confirmed for all seven 2018 (plasmid-free) 7PET-T13 genomes cultured at IP (Table S2). Similarly, some 2019 isolates were found resistant to doxycycline

(n=11) or tetracycline (n=2), or showed resistance to ciprofloxacin (n=17), but none of these phenotypes could be supported by genotype predictions with Abricate, nor were they confirmed when cultured at the IP (YE-NCPHL-19005-PI and YE-NCPHL-19008-PI are TET^S, YE-NCPHL-19009-PI is CIP^S; Table S2).

We thus consider that it is possible that reagent expiry at NCHPL may have contributed to the discrepancies between phenotypic and genotypic serotyping as well as ampicillin, tetracycline and ciprofloxacin resistance prediction.

On the other hand, the Abricate screen found that all 7PET-T13 Yemen isolates carried the *tet*(34) and *catB9* genes (Table S5) and were thus predicted to be resistant to tetracycline and chloramphenicol. However, *catB9* is known not to provide chloramphenicol resistance in this genomic background[8], consistent with chloramphenicol susceptibility of strains tested at IP (Table S3). Neither tetracycline resistance nor oxytetracycline resistance was observed in phenotypic tests conducted at NCHPL and IP (Tables S1, S2, S3), even though *tet*(34) has been described to only provide oxytetracycline resistance in a Mg^{2+}-rich medium[9], a condition that may not be met during the phenotypic tests. However, the gene *tet*(34) was described to be similar to the xanthine–guanine phosphoribosyl transferase gene of *V. cholerae*[10], and was detected in 881/882 of our assembled *V. cholerae* genome collection (Table S5), suggesting that it is a core gene of the species, and that any encoded tetracycline resistance phenotype would be shared across the species – which has never been observed; we therefore conclude that this *tet*(34) gene is a false positive hit not related to tetracycline or oxytetracycline resistance.

Given that serotype and antibiotic susceptibility test results obtained on the lower-size sample at the IP generally agreed with our genotype-based *in silico* predictions, we assumed NCHPL test results may have suffered from adverse conditions leading to unreliable results, and we therefore chose to report genotype-based predictions for these traits, except for false positive predictions of resistance to tetracycline, chloramphenicol.

Genomic context of the standalone MDR pseudo-compound transposon

In the ten assembled genomes of *V. cholerae* ST555 (*Vc*D) isolates, the MDR pseudo-compound transposon (PCT) YemVchMDRI is present without the IncC plasmid backbone observed in other genomic backgrounds of 2018-2019 Yemeni *V. cholerae* isolates. In the ST555 background, represented by the complete genome sequence of strain CNRVC190247 (= YE-NCPHL-18035-PI), the PCT is inserted in chromosome 2, between:
a) (in 5' of the PCT, referring to the orientation on pCNRVC190243) two transposases (CNRVC190247_02871 and CNRVC190247_02872), followed by a large ORF (CNRVC190247_02873) encoding a 1273-amino acids hypothetical protein, that is conserved among ST 555 genomes; the following segment begins with a gene encoding a protein of the NirD family (CNRVC190247_02875), which is part of the *V. cholerae* species core-genome;
b) (in 3' of the PCT, referring to the orientation on pCNRVC190243) a gene encoding a cobyric acid synthase (CNRVC190247_03766) and an operon coding for a molybdenum ABC

transporter (CNRVC190247_03763-03765); this region and the following segment are also part of the *V. cholerae* core genome.

In all Illumina short-read assemblies of ST555 genomes, YemVchMDRI appears as a standalone contig. Taking the genome assembly of strain YE-NCPHL-18060 (assembly id 33224_2#171) as an example, the entire contig #32 (length of 20,249 bp) corresponds to the PCT, except for the IS26 elements that should be located at both ends of the PCT (forming direct repeats). A scaffold assembly graph (based on the paired-end read information) however reveals that the PCT/contig #32 is connected at both ends to a contig which represents the IS26 element (contig #54), thus confirming the expected structure of the PCT. The IS26/contig #54 is itself connected to multiple contigs: contig #14 (106,332 bp) at one end, which corresponds to the region located upstream of the PCT on chromosome 2 of the reference strain CNRVC190247 genome; at the other end, through ramifications of the assembly graph, contig #54 is connected to contigs #11, #24, #29, #31, #36 and #45, none corresponding to regions located next to the PCT in reference strain CNRVC190247 genome, indicating that the short read-based assemblies cannot resolve the structure of this repeat-rich region. This pattern is replicated across all short-read assemblies of Yemeni ST555 strains. Despite uncertainty on what locus lies downstream of the PCT in short read-based assemblies of ST555 strains, the identity of the upstream regions with that of the hybrid assembly of reference strain CNRVC190247 provides reasonable support for the PCT being inserted at the same chromosomal locus in all Yemeni ST555 strains.

Phylogenetic hypothesis testing

From the ML tree (output of RAxML-NG) computed for the SNP alignment of 457 mapped 7PET genomes, some relationships between genome clusters appeared to be poorly-resolved (very low branch bootstrap supports) and indicated some alternative topologies would be equally supported. In particular, we considered the distribution of genomes in the initial ML tree to be not parsimonious with regard to the associated metadata. Indeed, a cluster gathering all genomes of isolates sampled from Kenya in 2010-2015 and Uganda in 2014-2016 (cluster *Vc*H.9.d in the final tree) was paraphyletic, due to the inclusion of a cluster of genomes of isolates sampled from Tanzania in 2015 and Zimbabwe in 2018 (cluster *Vc*H.9.c in the final tree). Similarly, the cluster containing most of the Yemeni isolate genomes from 2018 Yemeni isolates (cluster *Vc*H.9.h in the final tree) emerged from the clade containing most of the Yemeni isolate genomes from 2019 Yemeni isolates (cluster *Vc*H.9.g in the final tree). In both cases, the included clade was connected to the root of the inclusive clade by a string of branches with very low bootstrap supports. We therefore formulated hypotheses where the included clades (clusters *Vc*H.9.d and *Vc*H.9.h, respectively) would branch as sister clades of the initially inclusive clades (clusters *Vc*H.9.c and *Vc*H.9.g, respectively). We first tested the relationship between *Vc*H.9.h and *Vc*H.9.g clusters, and obtained nearly equal support for the inclusion of *Vc*H.9.h within VcH.9.g clade (log likelihood = -5719994.708326), or their branching as sister clades (log likelihood = -5719994.708058); the latter hypothesis received slightly higher support (Shimodaira-Hasegawa [SH] test *p*-values: 0.4629 and 0.5125, respectively) and was used as background for testing the relationship between *Vc*H.9.c and *Vc*VcH.9.d. We obtained

significantly stronger support for the branching as sister clades of *Vc*H.9.c and *Vc*H.9.d (log likelihood = -5719994.701928) when compared to the initial inclusion hypothesis (SH test *p*-values: 0.4871 and 0.0001, respectively). When the three hypotheses are compared together, the support is overwhelmingly in favour of the tree with both cluster pairs resolved as sisters (SH test *p*-values: 0.0001 for both hypotheses with *Vc*H.9.d included in *Vc*H.9.c, and 0.5167 for the sister-clade hypothesis); this latter tree topology is described in the main text and represented in Figure 2.

ICP1-like lytic bacteriophage

We detected the presence of an ICP1-family of lytic bacteriophage (ICP1) in 51/232 Yemeni *V. cholerae* genome assemblies, where they accounted for > 1% of sequencing reads per sample (Table S4). Occurrence of phage genomes in the sequenced genomic DNA from bacterial isolates likely reflects contamination of the isolate cultures with phages present in the original stool or sewage samples (see Methods). Some read sets showed high levels of contamination with phage-derived reads (16 samples with >50% ICP1 reads), suggesting that ICP1 phages may have infected the bacterial isolates in the samples and undergone several lytic cycles. The 51 assembled phage sequences showed an average 5.94 pairwise SNP differences (nucleotide diversity π of $4.87 \cdot 10^{-5}$), a variation two orders of magnitude lower than seen previously within a collection of diverse ICP1-family phages isolated over a 12-year period in Bangladesh[11]. The closest matches to the Yemeni ICP1 were found in the genome data for *V. cholerae* strain THSTI_45869, isolated in Delhi, India in 2015[12] (94% coverage and 99.7% nucleotide identity), and an ICP1 phage (MN402506.2) isolated in Chandigarh, India in 2015 from community sewage water (94% coverage and 99.9% identity; Chaudhary, N. and Taneja, N., direct submission to GenBank).

Phages were mostly found associated with *V. cholerae* O1-serogroup *Vc*H.9 strains (Figure S2A), consistent with the previously reported receptor specificity of this phage family[13], but were also associated with two non-O1/non-O139 *Vc*D strains. Presence of ICP1-like phage sequences in these genomic read sets (6.5% and 5% reads, respectively) suggests the phage was present and replicating in these samples, but it is possible that a bacterial strain mixture including an O1-serogroup strain occurred in the original samples, as was observed for other samples. ICP1-positive samples originated from five governorates, mostly from Al Mahwit and Sana'a (Tables S1, S4). The ICP1-like phage phylogeny showed limited geographic and temporal clustering, potentially reflecting a pattern of local transmission (Additional Supplementary Figure 2A). In addition, the viral phylogeny showed a branching structure incongruent with that of the bacterial host (Additional Supplementary Figure 2B, focusing on the *Vc*H.9 subtree), suggesting multiple horizontal transfers of the phage between host bacteria, consistent with the known lytic mode of transmission of ICP1-like phages.

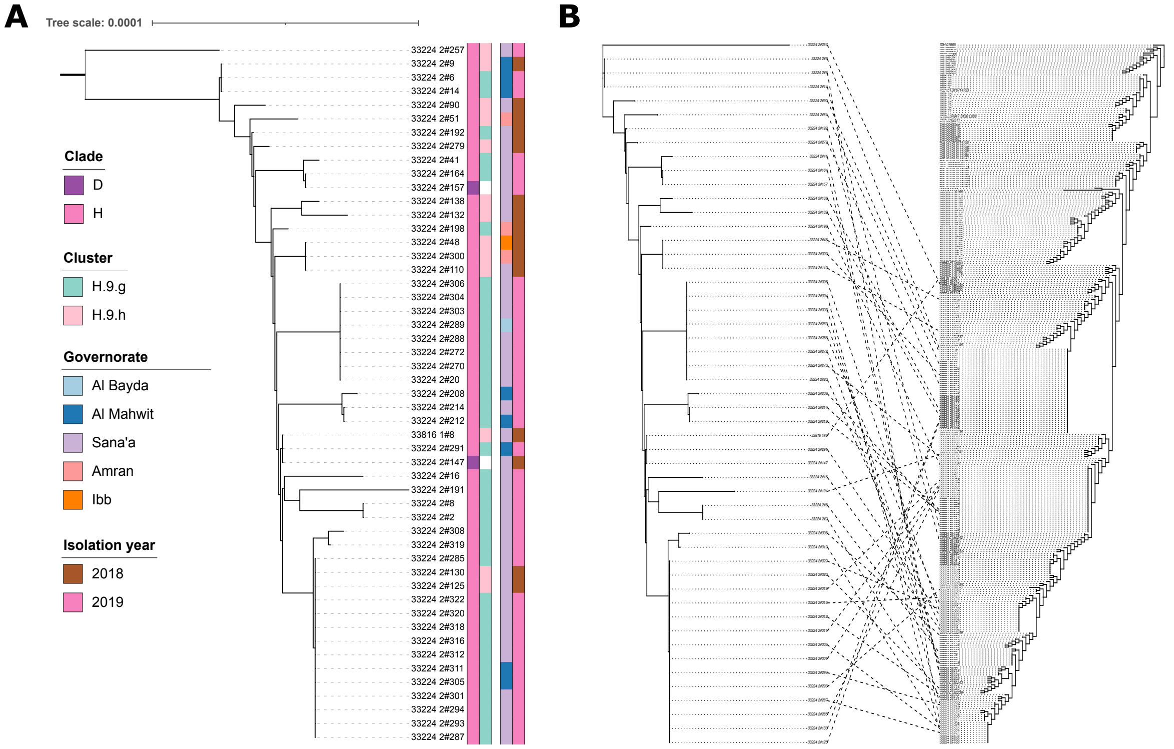

Additional Supplementary Figure 2: Phylogenetic diversity of ICP1-family phages from Yemen

A. Phylogeny of ICP1-family phage sequences present in Yemeni *V. cholerae* sequencing read sets. These sequences were assembled by mapping reads to the most complete phage sequence obtained from the genome assembly of sample YE-NCPHL-19021 (GenBank accession MW911613.1). Clade and phylogenetic clusters (when part of *Vc*H.9) of the host *V. cholerae* genomes, as well as year and governorate of collection of the samples are indicated in couloured strips on the right (see key). **B**. Tanglegram facing the host bacterial phylogeny on the left (mapped genome tree as presented in Figure 2A) and the viral phylogeny on the right, both represented as cladograms. Dashed links match host and viruses originating from the same sample.

Looking into genetic determinants of phage sensitivity that could explain variable phage occurrence in samples, we tested the significance of correlation of occurrence of the phage against presence of a range of known anti-phage defence systems or, without *a priori* targets, against all other pangenome genes, but found no significant association with any gene (see below). Instead the variable occurrence of the phage may result from the stochasticity of culture contamination during the process of bacterial isolation from the original samples. The recovery of an ICP-1-like phage in association with almost a quarter of *V. cholerae* isolates in Yemen in 2018-2019 suggests that this virus could often accompany the 7PET populations, as was previously observed in South Asia[11], and reinforces the view that these phages may represent biomarkers for 7PET *V. cholerae* populations[14].

Testing association of ICP1-like phages with other genomic traits

To investigate whether the variable presence of the ICP1 among Yemeni 7PET-T13 isolates could be explained by increased sensitivity or resistance conferred by some genetic determinants, we tested the significance of correlation of presence of the phage against occurrence of a range of other traits.

First, we looked into the association with the presence or absence of known anti-phage defense systems, none of which were significant: all *Vc*H genomes featured an intact ICEVchInd5 Hotspot 5 and none of the Yemeni host genomes carried the PICI-like elements (PLE) – both elements known to provide protection to the bacterial cell against bacteriophage infection or dissemination[15,16]. In addition, CRISPR-Cas loci were found in only four environmental Yemeni isolates (Table S5), carrying one or two loci of type I-F[17], all located within or next to a prophage locus. Three of these CRISPR-Cas-positive isolates belonged to *Vc*D (but outside ST 555; Table S5), one of which was ICP1-positive, and one (YE-NCPHL-SGTP0019002) belonged to 7PET-T13 cluster *Vc*H.9.g, and was not ICP1-positive. The latter occurrence is of particular interest as the presence of a CRISPR-Cas system has never been reported previously for 7PET isolates; in this 7PET isolate genome, the contigs carrying the CRISPR-Cas systems (contigs #28 and #29) both have landmarks of mobile elements such as integrases, suggesting they constitute a large Tn*7* transposon and a prophage, respectively, and show 100% homology with segments of larger contigs from genomes of *Vc*D isolates YE-NCPHL-SGTP0019009, suggesting these mobile elements may have been recently transferred from a *Vc*D background to a 7PET background. However, we cannot rule out that this occurrence of CRISPR-Cas-carrying MGEs in this 7PET isolate genome could stem from contamination by another genome, typically that of an endemic *Vc*D strain, or by the free form of the MGEs; occurrence of the same CRISPR-Cas-carrying elements in several other environmental sewage samples suggests such contamination could easily happen. Of note, the CRISPR-Cas-containing contigs of YE-NCPHL-SGTP0019002 genome are both only showing a single-end connection in the assembly graph (i.e. one end has no link to any other part of the genome based on paired-end read information), while the connected end links to a short repeated sequence; this again suggests these contigs were derived from standalone segments of DNA that did not belong to the strain's genome.

We further looked for other unknown anti-phage defence mechanism using correlation of pangenome genes against ICP1-like phage presence, but no gene – except the ICP1 phage's own genes – showed any strong pattern of (anti-)association with phage presence.

Ddm anti-plasmid defence systems

A recent study reported that two anti-plasmid defence systems, DdmABC and DdmDE, caused the instability of plasmids in *V. cholerae* cells, including those with IncC-type replicons[18]. These proteins are encoded by all 7PET genomes, which according to this study would explain the incapacity of plasmids to be maintained in a 7PET background. We here show that a 139-kb IncC-type plasmid has been stably propagated in a clone of the 7PET lineage. We verified the presence and integrity of the DdmABC and DdmDE systems and found they were present and intact in all 7PET genomes in our 882 assembled genome dataset – including those harbouring pCNRVC190243 (Table S5). This shows that these defence systems are not sufficient to destabilise pCNRVC190243 to the point of it being lost from the population within the 15-month period covered by our study, or even for the following two years, as suggested by 2021 antibiotic susceptibility profiles. This is consistent with data reported by Jaskólska et

al.[18], showing that Ddm is not sufficient to inhibit the colonisation of the *V. cholerae* cell by a large MDR IncC plasmid, although it confers a competitive disadvantage to plasmid-bearing cells[18]. This limited effect may become ecologically relevant as it can lead to loss of the plasmid from the population[18], but this effect is likely to be overcome in presence of selection for antibiotic resistance – a circumstance that occurred during the Yemen cholera outbreak due to large scale treatment of the population with drugs including azithromycin.

References

1. Hoshino, K. *et al.* Development and evaluation of a multiplex PCR assay for rapid detection of toxigenic Vibrio cholerae O1 and O139. *FEMS Immunol. Med. Microbiol.* **20**, 201–207 (1998).
2. von Mentzer, A. *et al.* Long-read-sequenced reference genomes of the seven major lineages of enterotoxigenic Escherichia coli (ETEC) circulating in modern time. *Sci. Rep.* **11**, 9256 (2021).
3. Dorman, M. J. *et al.* Genomics of the Argentinian cholera epidemic elucidate the contrasting dynamics of epidemic and endemic Vibrio cholerae. *Nat. Commun.* **11**, 4918 (2020).
4. Haley, B. J. *et al.* Genomic and Phenotypic Characterization of Vibrio cholerae Non-O1 Isolates from a US Gulf Coast Cholera Outbreak. *PLOS ONE* **9**, e86264 (2014).
5. Greig, D. R. *et al.* Evaluation of Whole-Genome Sequencing for Identification and Typing of Vibrio cholerae. *J. Clin. Microbiol.* **56**, (2018).
6. Wang, H. *et al.* Genomic epidemiology of Vibrio cholerae reveals the regional and global spread of two epidemic non-toxigenic lineages. *PLoS Negl. Trop. Dis.* **14**, e0008046 (2020).
7. Murase, K. *et al.* Genomic dissection of the Vibrio cholerae O-serogroup global reference strains: reassessing our view of diversity and plasticity between two chromosomes. *Microb. Genomics* **8**, 000860.

8. Weill, F.-X. *et al.* Genomic history of the seventh pandemic of cholera in Africa. *Science* **358**, 785–789 (2017).
9. Nonaka, L. & Suzuki, S. New Mg2+-Dependent Oxytetracycline Resistance Determinant Tet 34 in Vibrio Isolates from Marine Fish Intestinal Contents. *Antimicrob. Agents Chemother.* **46**, 1550–1552 (2002).
10. Roberts, M. C. Update on acquired tetracycline resistance genes. *FEMS Microbiol. Lett.* **245**, 195–203 (2005).
11. Angermeyer, A., Das, M. M., Singh, D. V. & Seed, K. D. Analysis of 19 Highly Conserved Vibrio cholerae Bacteriophages Isolated from Environmental and Patient Sources Over a Twelve-Year Period. *Viruses* **10**, 299 (2018).
12. Weill, F.-X. *et al.* Genomic insights into the 2016–2017 cholera epidemic in Yemen. *Nature* **565**, 230 (2019).
13. Seed, K. D. *et al.* Evidence of a Dominant Lineage of Vibrio cholerae-Specific Lytic Bacteriophages Shed by Cholera Patients over a 10-Year Period in Dhaka, Bangladesh. *mBio* **2**, (2011).
14. Nelson, E. J. *et al.* Gold Standard Cholera Diagnostics Are Tarnished by Lytic Bacteriophage and Antibiotics. *J. Clin. Microbiol.* **58**, (2020).
15. O'Hara, B. J., Barth, Z. K., McKitterick, A. C. & Seed, K. D. A highly specific phage defense system is a conserved feature of the Vibrio cholerae mobilome. *PLoS Genet.* **13**, e1006838 (2017).
16. LeGault, K. N. *et al.* Temporal shifts in antibiotic resistance elements govern phage-pathogen conflicts. *Science* **373**, (2021).
17. Abby, S. S., Néron, B., Ménager, H., Touchon, M. & Rocha, E. P. C. MacSyFinder: A Program to Mine Genomes for Molecular Systems with an Application to CRISPR-Cas Systems. *PLoS ONE* **9**, e110726 (2014).

18. Jaskólska, M., Adams, D. W. & Blokesch, M. Two defence systems eliminate plasmids from seventh pandemic Vibrio cholerae. *Nature* 1–7 (2022) doi:10.1038/s41586-022-04546-y.
