# Supplementary material for: Genomic epidemiology reveals multidrug resistant plasmid spread between Vibrio cholerae lineages in Yemen
Source: Nat Microbiol. 2023 Sep 28;8(10):1787–98. doi: 10.1038/s41564-023-01472-1 (PMC10539172; doi:10.1038/s41564-023-01472-1)
Supplement: Supplementary file 2 — Reporting Summary [file 41564_2023_1472_MOESM2_ESM.pdf]

nature portfolio

Corresponding author(s): Florent Lassalle, Ghulam Dhabaan, Nick Thomson

Last updated by author(s): Mar 3, 2023

Reporting Summary

Nature Portfolio wishes to improve the reproducibility of the work that we publish. This form provides structure for consistency and transparency in reporting. For further information on Nature Portfolio policies, see our Editorial Policies and the Editorial Policy Checklist.

Statistics

For all statistical analyses, confirm that the following items are present in the figure legend, table legend, main text, or Methods section.

n/a	Confirmed	
☐	☒	The exact sample size (n) for each experimental group/condition, given as a discrete number and unit of measurement
☒	☐	A statement on whether measurements were taken from distinct samples or whether the same sample was measured repeatedly
☐	☒	The statistical test(s) used AND whether they are one- or two-sided<br>*Only common tests should be described solely by name; describe more complex techniques in the Methods section.*
☐	☒	A description of all covariates tested
☒	☐	A description of any assumptions or corrections, such as tests of normality and adjustment for multiple comparisons
☐	☒	A full description of the statistical parameters including central tendency (e.g. means) or other basic estimates (e.g. regression coefficient) AND variation (e.g. standard deviation) or associated estimates of uncertainty (e.g. confidence intervals)
☐	☒	For null hypothesis testing, the test statistic (e.g. F, t, r) with confidence intervals, effect sizes, degrees of freedom and P value noted<br>*Give P values as exact values whenever suitable.*
☐	☒	For Bayesian analysis, information on the choice of priors and Markov chain Monte Carlo settings
☒	☐	For hierarchical and complex designs, identification of the appropriate level for tests and full reporting of outcomes
☐	☒	Estimates of effect sizes (e.g. Cohen's d, Pearson's r), indicating how they were calculated

Our web collection on statistics for biologists contains articles on many of the points above.

Software and code

Policy information about availability of computer code

Data collection	no software was used for data collection
Data analysis	genome assembly: SPAdes v3.10.0, UniCycler v0.4.7 and v0.4.8, pilon v1.23; genome annotation: Prokka version v1.5.0; sequence similarity searches: NCBI BLAST+ v2.7.1, Abricate v1.0.1, MacSyFinder v2.1, CRISPRCasFinder v1.1.2, ARIBA v2.14.6+, samtools/bcftools v1.9; phylogenetic analysis: RAxML-NG v1.0.1, Pantagruel version 8f95544, ClonalFrameML v1.11, BactDating v1.1; pangenome analysis: Panaroo82 v1.2.3; statistical analysis: R with packages 'ade4' and 'stats', custom code in https://github.com/flass/yemenpaper; geographical maps: QGIS 3.16.3.

For manuscripts utilizing custom algorithms or software that are central to the research but not yet described in published literature, software must be made available to editors and reviewers. We strongly encourage code deposition in a community repository (e.g. GitHub). See the Nature Portfolio guidelines for submitting code & software for further information.

Data

Policy information about availability of data

All manuscripts must include a data availability statement. This statement should provide the following information, where applicable:

- Accession codes, unique identifiers, or web links for publicly available datasets
- A description of any restrictions on data availability
- For clinical datasets or third party data, please ensure that the statement adheres to our policy

Short-read genomic data sequenced at the WSI were deposited at the ENA under the BioProject PRJEB34436. Four of the resulting assemblies comprised a single 123-kb contig corresponding to the ICP1-like phage; these assemblies were deemed uncontaminated and complete ICP1-like phage genomes and were deposited to GenBank under the accessions MW911612-MW911615. Complete hybrid genome assemblies for reference strains CNRVC019243 and CNRVC019247 were deposited to the ENA under the BioProject accessions PRJEB52123 and PRJEB47951 (Assemblies GCA_937000105 and GCA_937000115), respectively. Supplementary data are available online on the Figshare repository, under the following digital object ientifiers (doi): https://doi.org/10.6084/m9.figshare.16595999, https://doi.org/10.6084/m9.figshare.16611823, https://doi.org/10.6084/m9.figshare.18304961, https://doi.org/10.6084/m9.figshare.19097111, https://doi.org/10.6084/m9.figshare.19519105, https://doi.org/10.6084/m9.figshare.23653971, https//:doi.org/10.6084/m9.figshare.23849034.

Human research participants

Policy information about studies involving human research participants and Sex and Gender in Research.

Reporting on sex and gender	this section is not relevant as the study did not focus on the human aspect of cholera patients but on the bacterial pathogen; no human material was collected or studied.
Population characteristics	this section is not relevant as the study did not focus on the human aspect of cholera patients but on the bacterial pathogen; no human material was collected or studied.
Recruitment	this section is not relevant as the study did not focus on the human aspect of cholera patients but on the bacterial pathogen; no human material was collected or studied.
Ethics oversight	No ethics were required as the samples were not human material

Note that full information on the approval of the study protocol must also be provided in the manuscript.

Field-specific reporting

Please select the one below that is the best fit for your research. If you are not sure, read the appropriate sections before making your selection.

☐ Life sciences ☐ Behavioural & social sciences ☒ Ecological, evolutionary & environmental sciences

For a reference copy of the document with all sections, see nature.com/documents/nr-reporting-summary-flat.pdf

Ecological, evolutionary & environmental sciences study design

All studies must disclose on these points even when the disclosure is negative.

Study description	Among the clinical samples collected from suspected cholera patients in Yemen in from 2016 to 2019, presence of Vibrio cholerae was tested by microbiological culture and upon positive identification antibiotic susceptibility was performed on isolates. The changes in the antibiotic susceptibility pattern between 2018 and 2019 of these isolates prompted us to randomly choose 260 V. cholerae isolates from both years for whole genome sequencing (WGS) towards a genomic epidemiology analysis.
Research sample	260 V. cholerae isolates were chosen for WGS. 250 isolates were derived from clinical samples chosen randomly among the 4,375 samples confirmed to be positive for V. cholerae O1 by culture in 2018 and 2019 in Yemen. 10 additional isolates were derived from environmental samples obtained from the sewer system in Sana'a in 2019.
Sampling strategy	No calculation were done to establish adequate sample size. Sample size was determined due on the limited resources at the NCPHL lab in Sana'a in the context of the ongoing war and humanitarian crisis.
Data collection	Metadata related to the clinical samples were collected through the Electronic Disease Early Warning System (eDEWS), a surveillance programme coordinated by the Ministry of Public Health and Population of Yemen (MPHP) in Sana'a used to monitor the epidemic.
Timing and spatial scale	Samples sent for WGS were chosen randomly among a collection of samples obtained throughout the outbreak in 2018 and 2019, with spatio-temporal density of the sample roughly reflecting the variations in intensity of the outbreak through time and space. These samples originated from eight of the 21 Yemen governorates, comprising 71 out of 333 districts (Table S1), with 101 samples collected in 2018 (from mid-July to late October) and 149 in 2019 (from late February to late April and from early August to mid-October). In addition, ten environmentally-derived strains were isolated from sewerage in Sana'a in October 2019.

Data exclusions	Poor genome assemblies were filtered out if differing of more than 20% from the expected genome size of 4.2 Mb, or when more than 10% of reads were assigned by Kraken to another organism than V. cholerae (notably including the Vibrio phage ICP1) or to synthetic constructs, or were unclassified. This led to the omission of 28 genome assemblies, resulting in 232 high-quality assembled genomes to be included in the 882 assembled V. cholerae genomes dataset. Mapped genomes with an average read depth below 5x over the two chromosomes were deemed of insufficient read depth and were excluded (12 read sets mapped to CNRVC190243, all from this study and generated at WSI, were excluded for a final set of 456 mapped 7PET genomes; no read set mapped to CNRVC190247 was excluded).
Reproducibility	20 samples were sequenced twice, once at the Wellcome Sanger Institute (WSI; Hinxton, UK) and once at the Institut Pasteur (IP; Paris, France). For the isolates derived from these 20 samples, antibiotic susceptibility testing (AST) was also done twice, once at the National Centre of Public Health Laboratories (NCPHL; Sana'a, Yemen), once at IP. Discrepancies of outcome occurred, as 4 genomes derived from the same original sample were of different genotype; we explained this by the presence of multiple V. cholerae strains within the samples, an hypothesis confirmed by PCR testing of the samples. These isolates with distinct genotypes obtained from the same samples were then treated as separate isolates in downstream analyses.
Randomization	This is not relevant to our study, as groups of bacterial isolates were determined based on their genotype using phylogenetic analysis.
Blinding	The phylogenetic trees were initially drawn without any geographic information associated with the genomes.

Did the study involve field work? ☐ Yes ☒ No

Reporting for specific materials, systems and methods

We require information from authors about some types of materials, experimental systems and methods used in many studies. Here, indicate whether each material, system or method listed is relevant to your study. If you are not sure if a list item applies to your research, read the appropriate section before selecting a response.

Materials & experimental systems

n/a	Involved in the study	
☒	☐	Antibodies
☒	☐	Eukaryotic cell lines
☒	☐	Palaeontology and archaeology
☒	☐	Animals and other organisms
☒	☐	Clinical data
☒	☐	Dual use research of concern

Methods

n/a	Involved in the study	
☒	☐	ChIP-seq
☒	☐	Flow cytometry
☒	☐	MRI-based neuroimaging
